# Wogonin attenuates nasal polyp formation by inducing eosinophil apoptosis through HIF-1α and survivin suppression

**DOI:** 10.1038/s41598-018-24356-5

**Published:** 2018-04-18

**Authors:** Roza Khalmuratova, Mingyu Lee, Ji-Hun Mo, YunJae Jung, Jong-Wan Park, Hyun-Woo Shin

**Affiliations:** 1 Obstructive Upper airway Research (OUaR) Laboratory, Department of Pharmacology, Seoul National University College of Medicine, Seoul, Korea; 2 Department of Biomedical Sciences, Seoul National University College of Medicine, Seoul, Korea; 3 Department of Otorhinolaryngology-Head and Neck Surgery, Dankook University College of Medicine, Cheonan, Korea; 4 Department of Microbiology, School of Medicine, Gachon University, Incheon, Korea; 5 Ischemic/hypoxic disease institute, Seoul National University College of Medicine, Seoul, Korea; 6 Cancer Research Institute, Seoul National University College of Medicine, Seoul, Korea; 7 Department of Otorhinolaryngology-Head and Neck Surgery, Seoul National University Hospital, Seoul, Korea; 8 Clinical Mucosal Immunology Study Group, Seoul, Korea; 9 Gachon Advanced Institute for Health Science & Technology, Gachon University, Incheon, Korea

## Abstract

Chronic rhinosinusitis (CRS) with nasal polyps (CRSwNP) is an inflammatory sinonasal disorder characterized by eosinophilic inflammation and T-helper 2 skewing. Eosinophil accumulation in sinonasal mucosa comprises a major feature of CRSwNP. The study aimed to investigate the effect of the flavone wogonin in nasal polyposis by assessing its ability to induce eosinophil apoptosis *in vitro* and attenuate eosinophilic CRSwNP in mice. Double immunofluorescence, immunohistochemistry, flow cytometry, and immunoblotting were performed to evaluate hypoxia-inducible factor (HIF)-1α, survivin, and apoptotic markers in the human eosinophilic EoL-1 cell line or sinonasal tissues from patients with CRS with or without NPs. In sinonasal specimens from patients with CRS, HIF-1α and survivin were up-regulated in eosinophils from patients with NPs compared with levels in patients without NPs. Under hypoxia, HIF-1α and survivin expression was up-regulated in EoL-1 cells. Wogonin down-regulated both HIF-1α and survivin in EoL-1 cells. In addition, overexpression of survivin protected EoL-1 cells against apoptosis in response to wogonin. Moreover, wogonin attenuated nasal polyp formation in a murine model. Our findings suggest that wogonin could induce caspase-3 activation by suppressing HIF-1α and survivin expression in EoL-1 cells. Further studies regarding novel therapeutic options for CRSwNP targeting eosinophil apoptosis are needed.

## Introduction

Chronic rhinosinusitis (CRS) is a highly prevalent and heterogeneous disorder, characterized by chronic inflammation of the nasal cavity and paranasal sinus mucosa. CRS management remains a significant health concern worldwide and negatively affects the patient’s quality of life^1,2^. CRS is currently classified into two types: CRS with (CRSwNP) and without (CRSsNP) nasal polyps. Nasal tissue eosinophilia is considered as a major pathological hallmark of CRSwNP, particularly in Western populations^3^. However, recent studies support an increasing tendency for the prevalence of eosinophilic CRSwNP in Asian countries as well^4,5^. Furthermore, increasing evidence suggest that eosinophilic inflammation is correlated with polyp recurrence^6,7^. These findings support a role for eosinophils in CRSwNP, although the pathophysiology of this disease remains incompletely understood.

Persistent eosinophilic inflammation is related to the accumulation and prolonged survival of eosinophils in inflamed sinonasal tissue. At a site of inflammation, eosinophil survival increases to several days as a result of delayed apoptosis induced by various environmental factors and pro-inflammatory cytokines^8^. In addition, evidence exists that IL-5 and TNF-α promote eosinophil survival *in vitro*^9^. Activation of hematopoiesis within the bone marrow after allergen challenge may also contribute to the development and maintenance of a tissue eosinophilia^10^.

According to a study by Nissim *et al*.^11^, hypoxia-inducible factor (HIF)-1α prolongs the life of oxygen-deprived human eosinophils. It has been demonstrated that eosinophils respond to hypoxia by up-regulating HIF-1α and Bcl-XL. More recently, Baek *et al*.^12^ reported the influence of hypoxia on levels of airway inflammation and remodeling *in vivo*. The combined hypoxia and allergen enhanced HIF-1α expression and increased eotaxin-1, peribronchial eosinophils, and degree of airway remodeling (fibrosis) compared to either stimulus alone. These findings indicate that hypoxia may cause a profound effect on eosinophil function. However, little is known about the direct link between eosinophils and hypoxia in CRS.

Wogonin (5,7-dihydroxy-8-methoxyflavone) is one of the active components of flavonoids that are present in extracts from *Scutellariae radix*, commonly known as the Chinese herb “Huang Qin”. Recently, neuroprotective, anticancer, antiviral, and anti-inflammatory effects of wogonin have been discovered^13–16^. Wogonin was found to induce eosinophil apoptosis and have potential as an eosinophil apoptosis-inducing anti-inflammatory agent in allergic asthma^17,18^. Although apoptosis represents an important component for the resolution of inflammation, limited research has been performed related to eosinophil apoptosis in chronic disease. Therefore, we aimed to investigate the ability of the flavone wogonin to induce eosinophil apoptosis *in vitro* and attenuate nasal polyp formation in a mouse model of CRS exposed to ovalbumin (OVA)/Staphylococcal enterotoxin B (SEB).

## Methods

### Human subjects

All subjects were enrolled after providing written informed consent under the Dankook University Hospital Review Board-approved protocol (no 2012-11-008), and all research was performed in accordance with relevant guidelines/regulations. The diagnosis of CRS with or without nasal polyps (NP) was based on historical, endoscopic, and radiographic criteria. The diagnosis of sinus disease was based on history, clinical examination, nasal endoscopy, and computed tomography of the paranasal sinuses. Endoscopic sinus surgery was performed when patient symptoms and radiographic findings did not resolve at least 6 weeks after patients were treated with antibiotics, topical corticosteroids, decongestants, and/or mucolytic agents. Antibiotics and topical steroids were discontinued 14 days before surgery. The patients with a deviated nasal septum were considered as the control group. Subject characteristics are shown in Table 1. Tissues from uncinate process (UP) were obtained from controls (n  = 6) and CRS without nasal polyps (CRSsNP, n  = 13). NPs and UPs were obtained from CRS with NP (CRSwNP, n  =  27).

**Table 1:** Characteristics of study subjects.

	Control	CRSsNP	CRSwNP	
Total no. of subjects	6 (3 male)	13 (10 male)	27 (22 male)	19 (16 male)
Tissue used	UP	UP	UP	NP
Age (y), mean (SD)	54 (20)	41 (18)	46 (16)	44 (13)
Asthma, no.	0	1	3	2
Aspirin sensitivity, no.	0	0	0	0
Lund-Mackay CT score, mean (SD)	0 (0)	7.2 (4.9)	11.6 (3.9)	12.1 (3.7)
Blood eosinophils (cells/mm³)	170 (115.4)	208.4 (162.7)	319.6 (273.2)	328.9 (264)

### Immunofluorescence staining

Immunofluorescence staining was performed to demonstrate eosinophil major basic protein (EMBP), HIF-1α, survivin, and caspase-3 in sinonasal tissue samples. For complete details, see the Methods section of the online supplement.

### Cell lines and cell culture

EoL-1 (human eosinophilic), THP-1 (human monocytic), HMC-1 (human mast), and RPMI2650 cell lines, derived from squamous cell carcinoma of nasal septum, were obtained from the Korean Cell Line Bank (Seoul, Korea). EoL-1, THP-1, and RPMI2650 cell lines were cultured in RPMI-1640 supplemented with 10% heat-inactivated fetal bovine serum, 100 U/mL penicillin, and 100 μg/mL streptomycin, respectively. The HMC-1 cells were grown in Iscove’s Modified Dulbecco’s medium containing 10% FBS, 100 U/mL penicillin, and 100 μg/mL streptomycin. Cells were incubated in a humidified atmosphere at 37 °C under 20% O_2_/5% CO_2_ for normoxia or 1% O_2_/5% CO_2_ hypoxia. The cells were treated with various concentrations of wogonin, YC-1, and chaetocin. Wogonin was dissolved in dimethylsulfoxide (DMSO), diluted in phosphate buffered saline (PBS), and administered in culture medium (final concentration of DMSO was 0.5%). An equal volume of DMSO was added to control wells.

### Western blot analysis

Proteins were separated by 8–12% sodium dodecyl sulfate polyacrylamide gel electrophoresis and transferred to from the gels onto polyvinylidene difluoride (PVDF) membranes (Immobilon-P, Millipore, Bedford, MA). Membranes were blocked with a Tris/saline solution containing 5% skim milk and 0.1% Tween-20 and the incubation process was conducted with anti-HIF-1α(1:1000), anti-caspase-3 (1:1000), anti-PARP (1:1000; Cell Signaling Technology, Beverly, MA), and anti-survivin (1:1000) antibodies. After incubation, the membrane was washed three times for 30 minutes and then treated with peroxidase-conjugated anti-mouse or anti-rabbit IgG (Vector Laboratories, Burlingame, CA) for 1 hour. The membranes were developed using a chemiluminescent reagent (ECL; Amersham Life Sciences, GE Healthcare) and subsequently exposed to chemiluminescent film to visualize the proteins.

### siRNA and plasmid DNA transfection

Cells at 30% confluence were transfected with siRNAs using RNAiMAX reagent from Invitrogen (Carlsbad, CA) or with plasmids using Lipofectamine 2000 from Invitrogen.

### Annexin V and propidium iodide staining and flow cytometry

EoL-1, THP-1, RPMI2650, and HMC-1 cells were treated with wogonin (100 μM) and incubated under normoxic (21%O_2_) or hypoxic (1%O_2_) conditions for 12 h at 37 °C. Cell death was analyzed using the Annexin V FITC apoptosis detection kit (BD Biosciences, San Jose, CA) and flow cytometry. Flow cytometric analysis was immediately performed in a BD FACS Canto Flow Cytometer.

### Animals

All animal experiments were approved by the Institutional Animal Care and Use Committee of Seoul National University and were performed under strict governmental and international guidelines on animal experimentation. Sixty male BALB/c mice, weighing approximately 20 to 25 g, were divided into groups: OVA/SEB untreated group (n = 16), OVA/SEB-challenged group with intranasal administration of wogonin (n = 16), and OVA/SEB-challenged group treated with dexamethasone (n = 16). Control animals received phosphate-buffered saline (PBS) only (n = 12). For complete details, see the Methods section of the online supplement.

### Quantitative real-time RT-PCR

Quantitative real-time PCR analysis was utilized to evaluate mRNA expression levelf of eosinophil cationic protein (ECP), IL-4, IL-13, IFN-γ, IL-17A, CXCL1, CXCL2, CCL11, CCL24, T-box transcription factor (T-bet), GATA3, and ROR-related orphan receptor γ (ROR-γ) in mouse tissue samples. For complete details, see the Methods section of the online supplement.

### Immunohistochemistry

Survivin and collagen type I were immunostained in paraffin embedded sections. For complete details, see the Methods section of the online supplement.

### Statistical analyses

Data are expressed as the means ± SD. A non-parametric Mann-Whitney *U*-test was used to compare differences between groups. Statistical analyses and data plotting were performed using SigmaPlot (version 10, Richmond, CA). A value of *P* < 0.05 was considered to indicate statistical significance.

## Results

### HIF-1α and survivin expression in CRSwNP eosinophils

**Figure 1:**
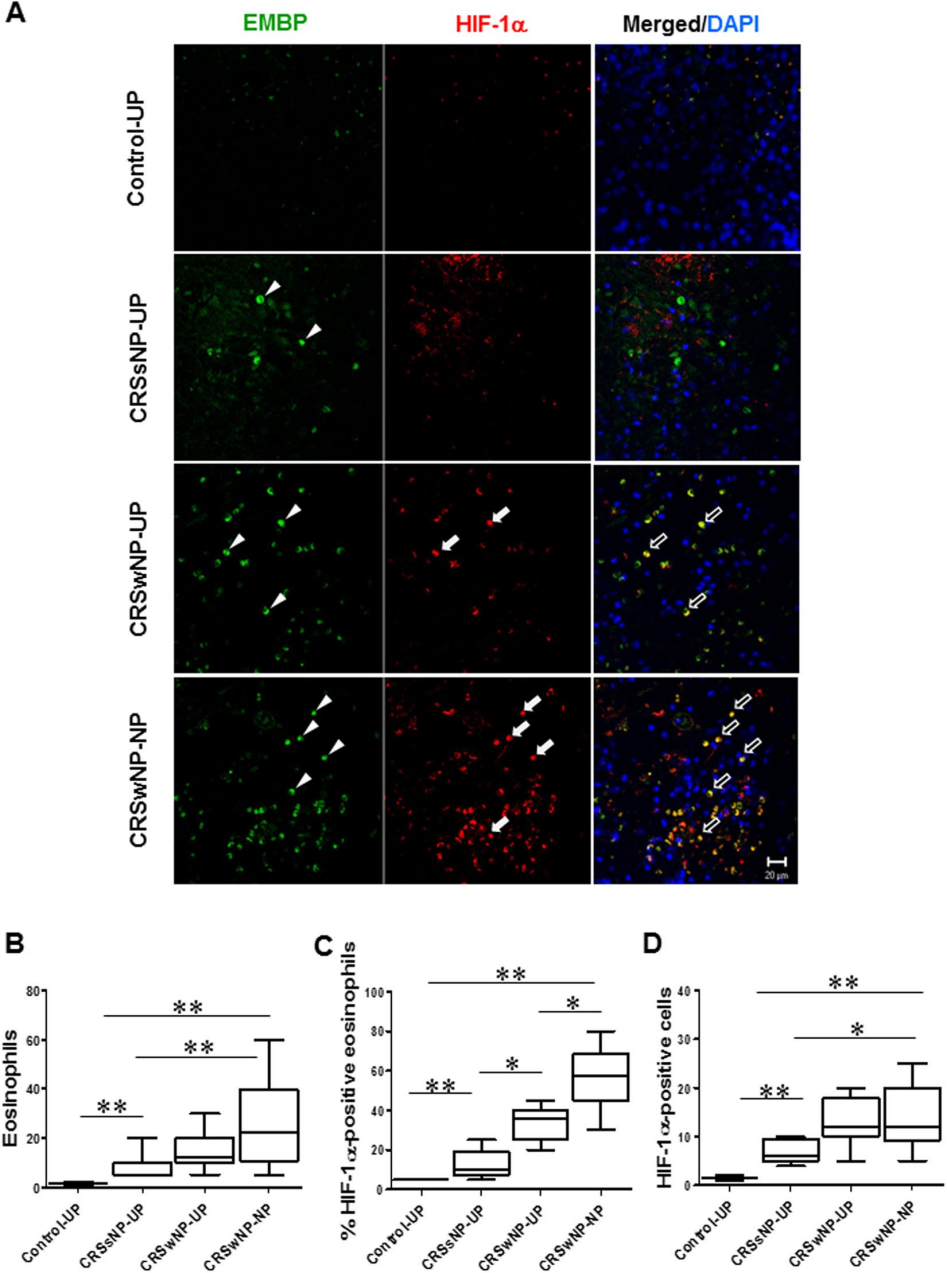
Fluorescence micrographs of HIF-1α expression and distribution of EMBP in nasal mucosal tissue. (**A**) Confocal images show colocalization of HIF-1α and EMBP (black arrows) in nasal mucosal tissue from control subjects and patients with CRSsNP, a patient with CRSwNP, and a NP sample. White arrowheads indicate EMBP-positive and arrows show HIF-1α-positive cells. Cell nuclei were counterstained by DAPI (4′,6-diamidino-2-phenylindole). (**B**–**D**) Distribution of eosinophils and percentage of HIF-1α-positive cells in nasal mucosal tissue. The number of HIF-1α- positive cells was considerably higher in the NP tissues of the CRSwNP group compared to the other groups. Data are expressed as the means ± SD. *P < 0.05, **P < 0.01. Mann-Whitney *U* test. Scale bar = 20 μm.

Tissue samples were collected from patients with CRSsNP (UP tissues), patients with CRSwNP (NP and UP tissues), and healthy subjects (UP tissues) to evaluate HIF-1α expression levels (Table 1). A significant increase in HIF-1α-positive immune cell numbers were observed in patients with CRSwNP (NP and UP tissues) (Fig. 1A). Moreover, HIF-1α expression was higher in EMBP-positive cells of NPs compared with that from patients with CRSsNP and normal UT. The HIF-1α immunoreactivity was mainly found in the nuclei, whereas positive staining of EMBP was generally found in the cytoplasm (Fig. 1B–D).

### Wogonin induces caspase-dependent apoptosis in the EoL-1 eosinophil cell line *in vitro*

**Figure 2:**
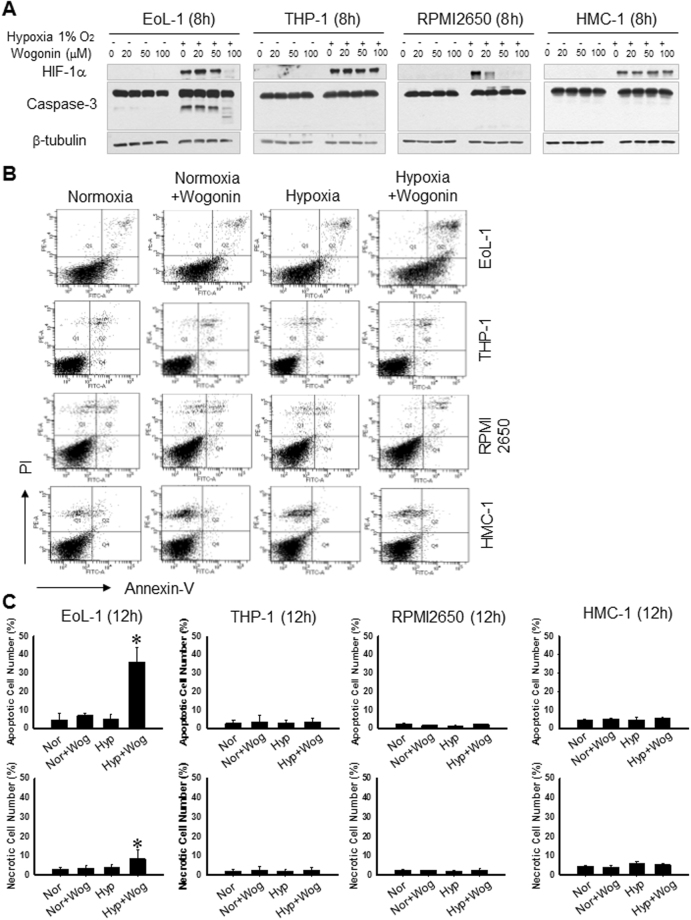
Wogonin induces concentration-dependent apoptosis in EoL-1 cells but not in THP-1, PRMI2650, and HMC-1 cells. (**A**) Representative western blots of HIF-1α, and cleaved caspase-3 in EoL-1, THP-1, RPMI 2650, and HMC-1 cells after wogonin treatment. Cells were treated with wogonin and incubated under normoxic (21% O_2_) or hypoxic (1% O_2_) conditions for 8 hours. Apoptosis was observed by detecting caspase-3 cleavage (17 kDa) using western blotting. (**B**) Cells were treated with 100 μM wogonin and incubated under normoxic or hypoxic conditions. Cells were stained with annexin-V and propidium iodide (Lou, #17) and subjected to flow cytometric analyses. (**C**) The number of cells in the above histograms were counted and plotted as bar graphs. Data are expressed as the means ± SD. *P < 0.05, **P < 0.01. Mann-Whitney *U* test.

EoL-1, THP-1, RPMI2650, and HMC-1 cells were incubated for 8, 16, and 24 hours with increasing concentrations of wogonin (0–100 μM) under normoxic and hypoxic conditions. As shown in Fig. 2A, HIF-1α expression levels increased in these cells under hypoxic conditions (Figure E1, and Fig. 1A–D). Treatment with wogonin decreased the expression of HIF-1α in EoL-1 and RPMI2650 cells in a concentration-dependent manner. Subsequently, apoptosis was analyzed by measuring caspase-3 activity and by flow cytometry. Treatment with wogonin (100 μM) resulted in induction of the cleaved form of caspase-3 in EoL-1 cells under hypoxia (Fig. 2A). We next examined the effect of wogonin on apoptotic death of EoL-1, THP-1, RPMI2650, and HMC-1 cells by flow cytometry (Fig. 2B and C); however, wogonin did not induce apoptosis in these cells. Thus, wogonin could induce eosinophil apoptosis in a cell type-specific manner. Conversely, YC-1 and chaetocin (HIF-1 inhibitors) showed cytotoxic effect in EoL-1 and THP-1 cell lines (Figure E2A and B).

### Inhibition of HIF-1α down-regulates the expression of survivin in EoL-1 cells

**Figure 3:**
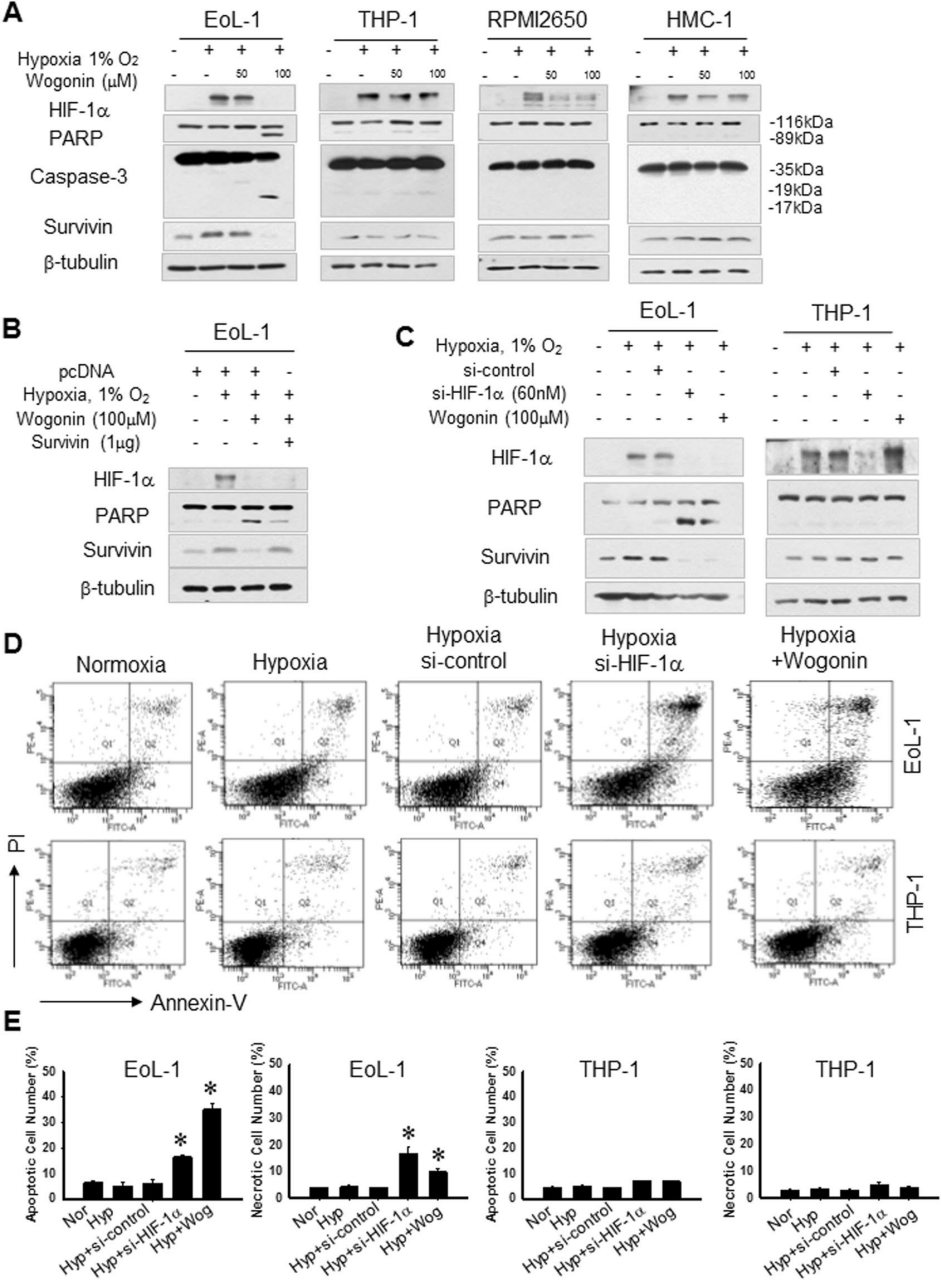
Inhibition of HIF-1α down-regulates survivin expression in EoL-1 cells. (**A**) Cells were treated with wogonin and incubated under normoxic or hypoxic conditions for 4 hours. HIF-1α, PARP, caspase-3 and survivin protein expression was detected. Survivin expression was up-regulated in EoL-1 cells under hypoxia. Wogonin decreases HIF-1α and down-regulates survivin expression in EoL-1 cells. (**B**) Survivin overexpression in EoL-1 cells delays cell apoptosis after wogonin treatment (**C**) EoL-1 and THP-1 cells transfected with 60 nM HIF-1α siRNA (si-HIF1α) or scrambled siRNA (Si-Cont) were incubated under normoxia or hypoxia for 8 hours. Cells were treated with 100 μM wogonin in hypoxic conditions. Cell lysates were prepared for immunoblotting with the indicated antibodies. (**D**,**E**) EoL-1 and THP-1 cells stained with annexin-V and propidium iodide were subjected to flow cytometry. Cells were treated with 100 μM wogonin.

As shown in Fig. 3A, survivin expression was upregulated in EoL-1 cells under the hypoxic condition, whereas wogonin-induced apoptosis was accompanied by a significant decrease of survivin. Moreover, we found that survivin overexpression protected EoL-1 cells against apoptosis in response to wogonin (Fig. 3B). As shown in Fig. 3C–E, in EoL-1 cells, wogonin-induced apoptosis was significantly increased after silencing HIF-1α. Collectively, our findings suggest that wogonin could induce caspase-3 activation by suppressing HIF-1α and survivin expression in EoL-1 cells.

### Immunohistological analysis of survivin expression in CRSwNP

**Figure 4:**
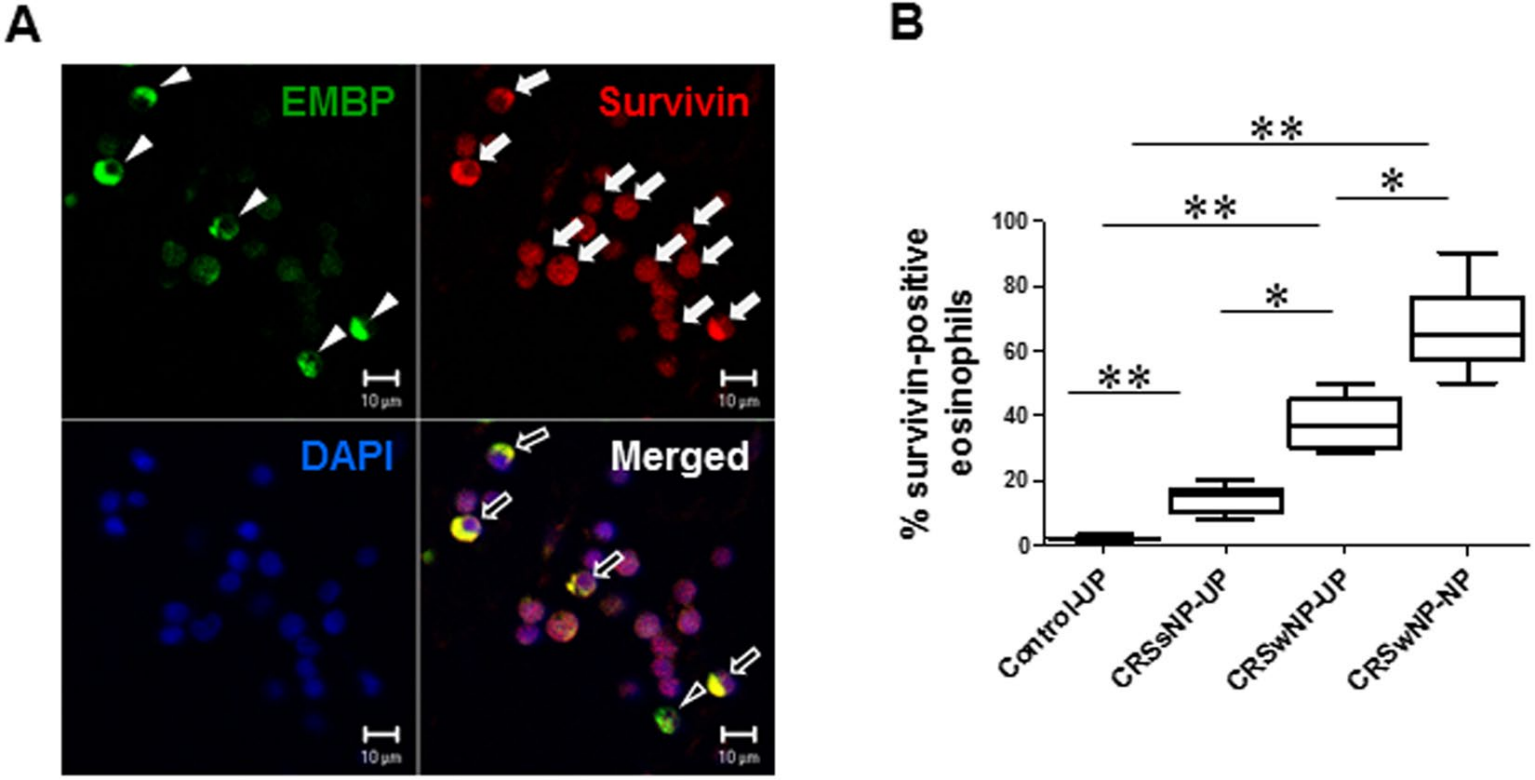
Double immunofluorescence micrographs of EMBP and survivin expression in human NP tissue. (**A**) EMBP-positive cells (arrowheads) support survivin expression. Survivin protein (white arrows) was dominantly expressed in the cell cytoplasm, unlike in the nucleus. (**B**) Percentage of survivin-positive eosinophils (black arrows) was significantly greater in the NP tissue. Data are expressed as the means ± SD. *P < 0.05, **P < 0.01. Mann-Whitney *U* test. Scale bar = 10 μm.

Obtained results revealed considerably higher numbers of survivin-positive cells in CRSsNP-UP tissues, whereas little or no survivin expression was observed in normal nasal mucosa. Survivin expression was elevated in NP mucosa from patients with CRSwNP compared with UP from control subjects and those with CRSsNP (Figure E3). Our finding demonstrate that human NPs had increased survivin expression in infiltrating immune cells. We then used double immunofluorescence staining to detect survivin-positive eosinophils in submucosa. We found a significant increase in survivin-positive eosinophil numbers in NPs and UPs of patients with CRSwNP compared with those in UPs from control subjects and patients with CRSsNP (Fig. 4A and B). In summary, these findings demonstrate that NPs had increased survivin expression in EMBP-positive cells.

### Wogonin reduces NP formation in BALB/c mice

**Figure 5:**
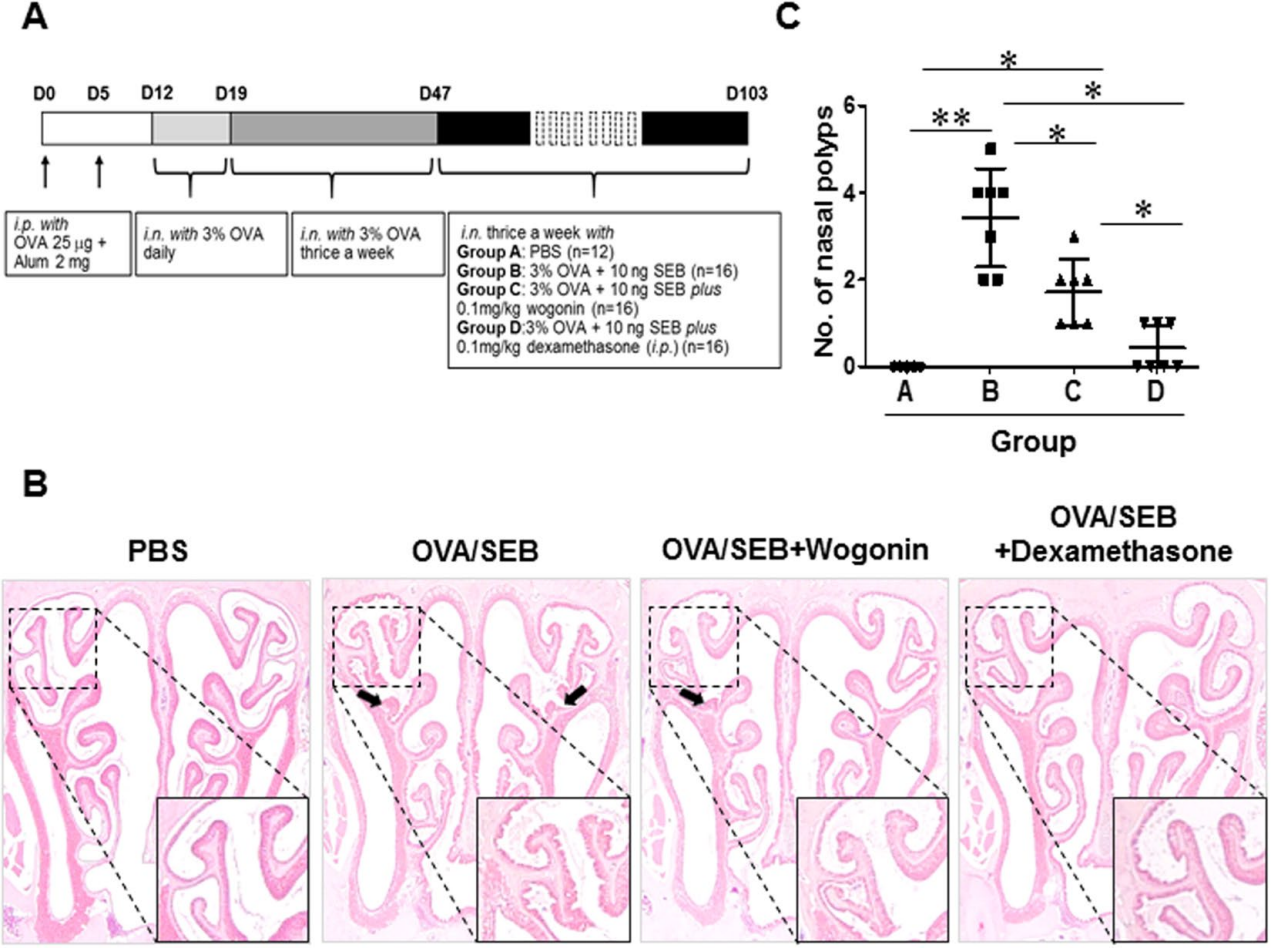
Nasal polyp burden is reduced in the wogonin-treated group. (**A**) Protocol for the development of chronic eosinophilic rhinosinusitis with nasal polyps in mice. Alum, Aluminum hydroxide; i.p., intraperitoneal injection; i.n., intranasal instillation. (**B**) and (**C**) Photographs of representative nasal polypoid lesions (arrows) stained with hematoxylin and eosin (original magnification ×1.25). Nasal polypoid lesions were detected in Groups B, C, and D. Numbers of nasal polyp-like lesions were expressed as the total number per coronal section. The numbers of polyp-like lesions were reduced in Groups C and D compared with that in Group B. The black box indicates the magnified area. Data are expressed as the means ± SD. *P < 0.05, **P < 0.01. Mann-Whitney *U* test. Scale bar = 20 μm.

All animals were treated with OVA and SEB to induce nasal polyps according to a previously described protocol (Fig. 5A). As shown in Fig. 5B, no polyp-like lesions were observed in the control group (received PBS; group A); polyp-like lesions were found only in the mice that received SEB intranasally (groups B, C, and D). A thickened mucosa with polyp-like lesions was observed primarily at the transition zone of the olfactory and respiratory epithelia. Mucosal polyp and epithelial disruption numbers in the nasal cavities were reduced in wogonin and steroid treated groups (Fig. 5C; Figure E4A and B).

### Double immunofluorescence staining of survivin and caspase-3 expression in the NP mouse model

**Figure 6:**
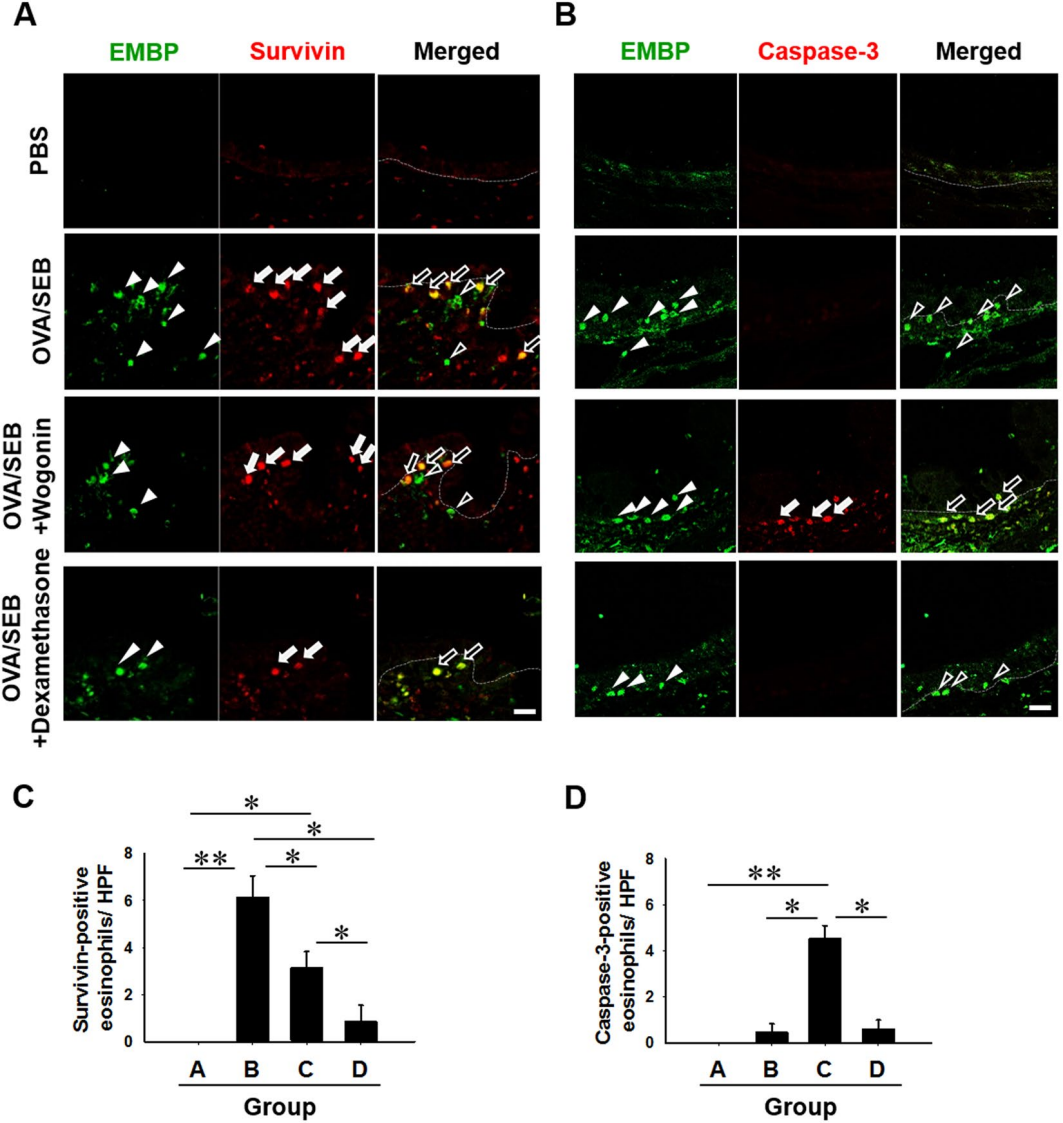
Fluorescence micrographs of EMBP, survivin, and caspase-3 positive cells in the mouse polyp model. (**A**) Representative photographs of survivin-positive and (**B**) caspase-3-positive eosinophils. (**C)** and (**D**) Quantitative analysis of survivin and caspase-3 -positive eosinophils. Dotted lines represent the border between respiratory epithelium and submucosa. Arrowheads denote EMBP-positive cells in each group of mice. White arrows denote survivin-positive cells in A and caspase-3-positive cells in B. Data are expressed as the mean ± SD. *P < 0.05, **P < 0.01. Mann-Whitney *U* test. Scale bar = 10 μm.

We used double immunofluorescence staining to detect survivin-positive immune cells in submucosa (Fig. 6A). NP models demonstrated a significantly increased survivin expression in immune cells compared with that observed in PBS-applied animals. In the wogonin-treated group, survivin-positive eosinophils were down-regulated compared to the untreated group (Fig. 6C). Moreover, caspase-3-positive eosinophils were detected in the wogonin-treated group, as shown in Fig. 6B and D.

### Alterations in cytokine and chemokine mRNA profiles after wogonin treatment

**Figure 7:**
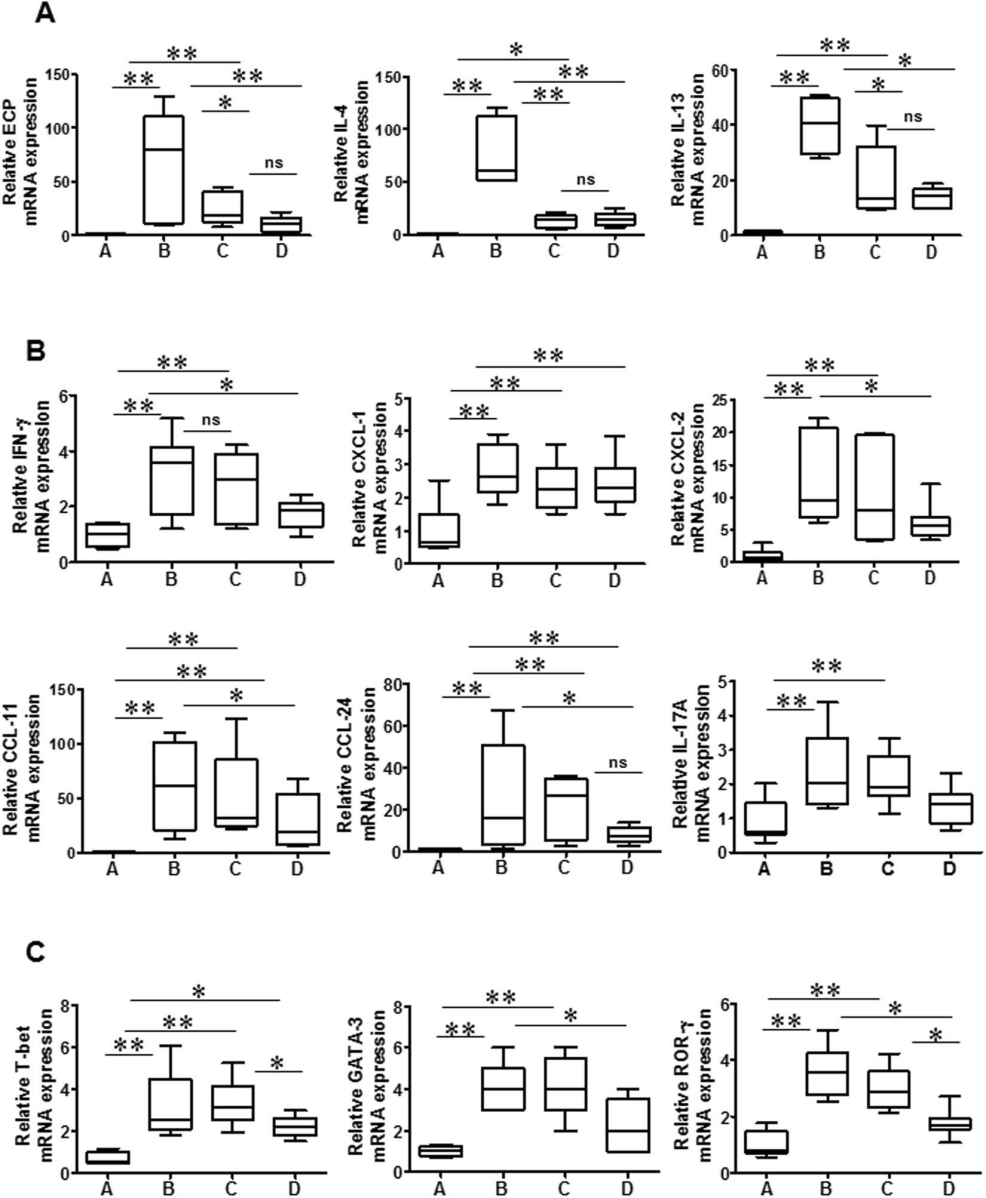
Wogonin suppresses mRNA expression of ECP, IL-4, and IL-13 in the mouse polyp model. (**A**–**C**) Relative mRNA expression levels of ECP, IL-4, IL-13, IFN-γ, IL-17A, CXCL1, CXCL2, CCL11, CCL24, T-bet, GATA3, and ROR-γ were measured in nasal tissues from the mouse polyp model. Groups A–D are defined as shown in Fig. 4. Data are expressed as the means ± SD. *P < 0.05, **P < 0.01. Mann-Whitney *U* test.

Next, we evaluated alterations in cytokine and leukocyte-recruiting chemokine mRNA profiles in NP mice. Wogonin treatment reduced the mRNA expression level of pro-inflammatory cytokines such as IL-4, IL-13, and ECP (Fig. 7A). Reduction in IFN-γ, IL-17A, neutrophil-recruiting chemokines (CXCL1 and CXCL2), eosinophil-recruiting chemokines (CCL11 and CCL24), T-bet, GATA3, and ROR-γ mRNA profiles were observed only in the steroid treated group (Fig. 7B and C). Thus, these data suggest that wogonin has anti-inflammatory properties related to the inhibition of cytokines secreted by eosinophils.

### Wogonin decreases eosinophil accumulation *in vivo*

We then analyzed the cellular composition of the nasal fluid of mice 24 hours after the last OVA challenge. Inflammatory cell levels in nasal fluid were significantly elevated in OVA/SEB challenged mice compared with those in control animals (Figure E5A–C). Intranasal wogonin administration led to a reduction in total inflammatory cells, with a concomitant increase in apoptotic cells that exhibited nuclear condensation and cellular shrinkage. Notably, the percentage of apoptotic eosinophils relative to total eosinophils was significantly higher in NP mice after wogonin treatment compared with that in untreated mice. The observed reduction in the recruitment of inflammatory cells into the upper airway correlated with the histopathological changes in nasal mucosa. These results indicated that treatment with wogonin efficiently decreased eosinophil accumulation and attenuated NP formation in mice. In addition, the total IgE and OVA-specific IgE was significantly reduced in the serum and nasal fluid samples in the wogonin-treated group compared to the untreated group (Figure E5D–G). Mice that received wogonin showed apoptotic bodies and caspase-3-positive cells in the submucosa. Moreover, survivin-positive cells were detected in the untreated group (Figure E6A–C).

### Effect of wogonin administration on inflammatory responses and airway remodeling

To investigate the effect of wogonin on the inflammation of nasal mucosa, histopathological examination was performed, which shows the changes of inflammatory and mucin producing cells. Both wogonin and steroid treatment significantly reduced the number of infiltrated eosinophils and goblet cells compared to the untreated group (Figure E7A–D). Histological analyses revealed that epithelial hyperplasia and maximal mucosal thickness were more pronounced in untreated mice compared with wogonin-treated mice. Furthermore, wogonin treatment decreased collagen type I deposition in submucosa (Figure E7E–G).

## Discussion

Eosinophils have cytotoxic functions and are involved in both the innate and adaptive immune responses^19^. Tissue eosinophilia increases the likelihood of recurrent disease and comorbid asthma in patients with CRSwNP, indicating that eosinophils play a central role in CRSwNP pathophysiology^20^. The severity of clinical symptoms of patients with allergies was reported to correlate with the number of eosinophils in the inflamed tissues^21^. Activated eosinophils release cytotoxic molecules such as major basic protein, eosinophil peroxidase, eosinophilic cationic protein, and lipid mediators that cause tissue damage^22^. Eosinophils are known to produce and release various proinflammatory cytokines^23^ such as IL-1β, IL-6, and TNF-α along with chemokines including IL-8/CXCL8, growth-regulated oncogene (GRO)-α/CXCL1, MIP-1β/CCL4, MCP-1/CCL2, and RANTES/CCL5^24^.

Several studies have demonstrated that hypoxia contributes to the pathobiology of CRS and NP formation^21^. High expression of HIF-1α in the inflammatory cells, fibroblasts, endothelium, glandular cells, and epithelium were observed in NPs by Hsu *et al*.^25^. In another study, Early *et al*.^26^ showed that hypoxia stimulates inflammatory and fibrotic responses from NP-derived fibroblasts. Moreover, the expression of the HIF-1α and HIF-2α was up-regulated in NPs and mediated nasal polypogenesis through the epithelial-to-mesenchymal transition^27^. However, the role of HIF-1α in nasal polyposis remained unclear^28^.

We investigated HIF-1α expression levels in eosinophils in NPs and compared these with healthy subjects, patients with CRSsNP-UP, and CRSwNP-UP. High levels of HIF-1α-positive eosinophils were observed in NP tissue. Notably, HIF-1α and survivin expression levels were up-regulated in EoL-1 cells under hypoxia. Moreover, we detected survivin-positive eosinophils in NP. Survivin-positive cells were also found in submucosa in mice. These latter findings are consistent with a previous report by Qie *et al*.^29^, which documented a markedly increased expression of survivin in NP at both the mRNA and the protein level. Thus, further studies are warranted to clarify the mechanisms responsible for eosinophil survival in hypoxic conditions.

Previous studies have suggested that survivin is essential for cell survival. The survivin protein contains structural features of the inhibitor of apoptosis protein (IAP) family. Active caspases can be suppressed by the IAP family to protect cells from activation of the caspase cascade^8,30^. Elevated expression of IAP in eosinophils has been correlated with cell survival and resistance to apoptosis under hypoxic microenvironment. Wogonin decreased the expression of HIF-1α and survivin, and finally induce the death of eosinophils in a caspase-dependent manner *in vitro* and *in vivo* models. Moreover, it has been reported that wogonin-induced apoptosis was also accompanied by a significant decrease of survivin along with Bcl-2 and increase of Bax in cancer cells^13^.

Here, we evaluated the role of wogonin administration in the context of resolution of allergic inflammation in a mouse model of CRS exposed to OVA/SEB. The results presented here can be summarized as follows: (i) treatment with wogonin reduced eosinophil accumulation in the nasal fluid and sinonasal tissue; (ii) wogonin promoted resolution of inflammation by inducing caspase-dependent apoptosis of eosinophils; (iii) treatment with wogonin decreased the inflammatory profile, mucus production, and collagen deposition.

In a recent study by Lucas *et al*.^17^, wogonin administration attenuated OVA-induced airway inflammation in the lung with reductions in bronchoalveolar lavage and tissue eosinophil numbers along with mucus production and increased eosinophil apoptosis. Another study by Lucas *et al*.^31^ demonstrated that wogonin was able to induce neutrophil apoptosis by down-regulating Mcl-1 and to enhance the resolution of neutrophilic inflammation *in vivo*. Recently Ryu *et al*.^14^ found that wogonin significantly inhibited allergen-induced eosinophilic inflammation in Balb/c mice, specifically by reducing the total IgE and OVA-specific IgE levels compared with the untreated group. Moreover, wogonin has been shown to have anti-fibrotic effect in the upper airway. These findings indicated that wogonin may inhibit TGF-β1-induced myofibroblast differentiation, extracellular matrix production, migration, and collagen contraction. Conversely, wogonin had no cytotoxic effects on TGF-β1-induced nasal-polyp-derived fibroblasts^15^.

Inteleukin (IL)-5 plays a key role in differentiation and activation of eosinophils. IL-5 has been found to be elevated in NP tissue and identified that IL-5 was a major eosinophil survival factor. Current therapeutic approach primarily target the adaptive immune response: anti-IL-5 treatment induced eosinophil cell death in an *ex vitro* polyp tissue model^32^. Mepolizumab (anti-human IL-5 monoclonal antibody) treatment result in a significant reduction of NP size in 50–60% patients^33^. Gevart *et al*. evaluated the effect of 2 intravenous injections of 750 mg of mepolizumab in patients with severe CRSwNP^34^. However, intranasal wogonin administration has advantages over intravenous and subcutaneous administration of current anti-eosinophil therapies such as an ease of use, efficacy and safety in long-term treatment.

Our observations are consistent with previous findings and provide solid evidence that wogonin may have potential therapeutic use for NP. Our discovery of a new mechanism regulating eosinophil function under hypoxia is notable because it contributes to the pathophysiology of NP. Furthermore, several pathways that modulate eosinophil survival or death constitute targets or proposed targets for future therapy for CRSwNP or related disorders of altered immune response.

## Electronic supplementary material

Supplementary information
