# Supplementary material for: Wogonin attenuates nasal polyp formation by inducing eosinophil apoptosis through HIF-1α and survivin suppression
Source: Sci Rep. 2018 Apr 18;8:6201. doi: 10.1038/s41598-018-24356-5 (PMC5906673; doi:10.1038/s41598-018-24356-5)
Supplement: Supplementary file 1 — Supplementary information [file 41598_2018_24356_MOESM1_ESM.pdf]

Wogonin attenuates nasal polyp formation by inducing eosinophil apoptosis through HIF-1α and survivin suppression

Roza Khalmuratova, MD, PhD,[1] Mingyu Lee, BSc,[1,2,6] Ji-Hun Mo, MD, PhD,[3,8] YunJae Jung, MD, PhD,[4,9] Jong-Wan Park, MD, PhD,[1,2,5,6] Hyun-Woo Shin, MD, PhD[1,2,5,6,7,8*]

Online Repository Methods

Immunofluorescence and confocal microscopy

For double-immunofluorescence staining, 4-μm tissue sections were deparaffinized in Neo-Clear (Merck KGaA, Darmstadt, Germany) and rehydrated using a series of ethanol washes. Epitope retrieval was performed by heating deparaffinized tissue sections in citrate buffer (10 mM sodium citrate, pH 6.0, 0.05% Tween 20) for 20 minutes at 98°C and cooling down in the same buffer for 20 minutes. After this and each subsequent immunostaining step, the sections were washed 3 times, 5 minutes each, in 1× PBS. Briefly, tissue sections were preincubated with PBS containing 0.1% Triton X-100 and 3% bovine serum albumin (BSA), the corresponding rabbit-anti-HIF-1α antibody (1:100; Novus Biologicals, Littleton, CO), rabbit-anti-survivin antibody (1:200; Novus Biologicals) and mouse anti-EMBP antibody (1:100; Santa Cruz Biotechnology) were applied and left overnight at 4°C. Tissue sections were then incubated in a mixture of Alexa -488 and Alexa-555 (1:300; Invitrogen) conjugated secondary antibodies diluted in blocking buffer for 2 hours at room temperature. Nuclei were counterstained with DAPI, 1 μg/mL, Sigma). Slides were mounted with fluorescence-mounting medium (Vectashield, Vector Labs, Burlingame, CA). Images were acquired with a Zeiss fluorescent microscope using AxioVision software (Carl Zeiss, Thornwood, NY). The HIF-1α immunoreactivity was mainly found in the nuclei, whereas positive staining of anti-EMBP and survivin was generally found in the cytoplasm.

For evaluation of the expression of EMBP, HIF-1α, survivin, and caspase-3-positive cells, in each case, two interval slides were selected and observed to evaluate immunopositive cells. The mean percentage of positive cells was calculated as positive cells/total cells × 100% in at least two areas using the ×40 or ×100 objective lens. The immunostaining was not observed in control sections incubated with normal serum instead of the primary antibodies (data not shown).

Animal model and tissue preparation

All animal experiments were approved by the Institutional Animal Care and Use Committee of Seoul National University and performed under strict governmental and international guidelines on animal experimentation. A total of 60 male BALB/c mice (4 weeks old, 20–25 g) were purchased from Central Laboratory Animal (Seoul, Korea) and housed for 1 week before initiating experiments. These animals were kept in a special pathogen-free biohazard containment facility maintained at 22–24°C and 50–60% humidity.

The experimental protocol was designed as described previously with some modifications (Fig 1, A)[1]. The mice were randomly categorized into one control (group A; n = 12) and three experimental groups (B, C, and D; n = 16 each).

The mice in the experimental group were systemically sensitized with 25 μg OVA (Grade V; Sigma) dissolved in 300 mL 1× PBS in the presence of 2 mg aluminum hydroxide gel adjuvant by intraperitoneal injection on Day 0 and Day 5. At seven days after the last immunization, mice were challenged intranasally with 3% OVA in 40 μL of 1× PBS daily for seven days. Intranasal instillation was performed in the head-down position, with the mouse head kept down for 30 seconds after instillation to prevent pulmonary provocation. Prolonged continuous inflammation was maintained in the experimental groups by the subsequent nasal challenge of mice to 3% OVA three times weekly for 12 consecutive weeks. Next, 10 ng of SEB (List Biological Laboratories, Inc., Campbell, CA) diluted in PBS were challenged immediately after the instillation of 3% OVA during the last 8 consecutive weeks (Group B). Wogonin (0.1 mg/kg; Sigma) was administered intranasally three times per week

from day 49 through day 102 before OVA instillation in Group C. Group D was intraperitoneally injected with dexamethasone (1 mg/kg/w). In the control group, PBS was applied for both systemic and local stimulation. Mice were sacrificed 24 hours after the last intranasal administration. The sinonasal specimens and nasal lavage were collected and processed according to the method previously described[1]. After partial tracheal resection, a micropipette was inserted into the choana through the tracheal opening in the direction of the upper airway. Each nasal cavity was gently perfused with 200 μL PBS and the fluid from the nostril was collected and centrifuged. The supernatants were stored at −80°C prior to use. Cell pellets were suspended in PBS and spun onto a slide. After the slides were dried, cells were fixed and stained using Diff-Quik stain (Dade Behring, Marburg, Germany) according to manufacturer instruction. The heads of mice from each group (n = 6 for control mice, n = 8 for OVA/SEB untreated group, n = 8 for OVA/SEB-challenged group with intranasal administration of wogonin, n = 8 OVA/SEB-challenged group treated with dexamethasone) were removed *en bloc* and then fixed in 4% paraformaldehyde for histopathologic analysis. After exposing the nasal cavities of the other mice, the nasal mucosa was taken out meticulously with a small curette and microforceps under microscopic vision.

Histopathological analysis of animal tissues

For evaluation of nasal histopathology, nasal tissues were decalcified, dehydrated, and processed according to standard paraffin-embedding procedures. The tissues were cut in coronal sections at 4-μm thickness. An atlas of normal murine sinonasal anatomy was used to standardize the anatomic locations being examined[2]. Several stains were utilized to compare characteristics between groups: hematoxylin and eosin (H&E) for polyp-like lesions, Sirius red for eosinophils, periodic acid-Schiff (PAS) stain for goblet cells, and Masson trichrome stain to measure subepithelial and epithelial thicknesses. We chose 10 areas from nasal mucosal sections randomly for evaluation under hpf (magnification ×1000) that were measured by 2 examiners who were blind to group assignment. Polyp-like lesions were defined as distinct mucosal elevations with eosinophilic infiltration and microcavity formation. Three consecutive slides were reviewed to exclude processing errors. Mucosal thickness was measured as the

distance between the apex of the epithelial cells and the upper border of the subepithelial glands zone by using an image analysis system. For assessment of mucosal thickness, at least 3 measurements at random points with a minimum distance of 20-μm between the points were made in the appropriate area of each hpf, and the mean from 4 different hpfs was recorded for comparison.

Immunohistochemical analysis

Immunohistochemistry was performed by using the polink-2 plus polymerized horseradish peroxidase (HRP) broad DAB Detection System (Golden Bridge International Labs., Mulkiteo, WA). Paraffin sections of nasal tissue were mounted on slides and dried at room temperature for 24 hours. The sections were deparaffinized, rehydrated, and autoclaved at 121°C for 10 minutes in 100 mmol/L citrate buffer (pH 6.0; Dako, Glostrup, Denmark) to retrieve antigens. After treatment with 3% hydrogen peroxide (Sigma) in methanol for 10 minutes, the sections were incubated in 3% BSA at room temperature for 1 hour to block nonspecific signals. Tissue sections were incubated against survivin (1:200), collagen type I (1:500, Abcam, Cambridge, MA) and neutrophil-elastase (1:200, Abcam) overnight at 4°C. The sections were incubated in broad antibody enhancer and polymer-HRP and then stained with the DAB Detection System. To standardize color development, the incubation time for diaminobenzidine staining was fixed in all experiments. Sections were counterstained with Gill's hematoxylin for 3 minutes and dehydrated through a graded ethanol series, cleared with xylene, and coverslipped with mountant. Negative controls were performed by omitting the primary antibody. Unless noted otherwise, all manufacturer instructions were followed. The slides were evaluated with a bright-field microscope (BX-51; Olympus, Tokyo, Japan) and a micrograph field of view of the entire stained section. The immunostaining was not observed in control sections incubated with normal serum instead of the primary antibodies (data not shown).

Detection of apoptotic cells with the TUNEL assay

Apoptotic cells were visualized using the TUNEL technique (Novus). Fixed tissue sections were deparaffinized, rehydrated, treated with proteinase K, and incubated with a terminal deoxynucleotidyl

transferase (TdT) enzyme solution at 37°C for 2 hours. The reaction was carried out at 37°C for 30 minutes and terminated in a stop and wash buffer provided by the supplier. The sections were incubated with anti-digoxigenin peroxidase and then with diaminobenzidine containing 0.01% H_2O_2 for 5 minutes. Finally, the sections were lightly counterstained with H&E and examined under a microscope. No staining was evident in negative controls when omitting the TdT enzyme. All of the procedures were performed according to the manufacturer's instructions.

Quantitative real-time reverse transcription-polymerase chain reaction (qRT-PCR) for inflammatory markers, chemokines, and transcription factors

Total RNA was isolated from the tissue samples (n = 6 for control mice, n = 8 for OVA/SEB untreated group, n = 8 for OVA/SEB-challenged group with intranasal administration of wogonin, and n = 8 for OVA/SEB-challenged group treated with dexamethasone) by using the RNeasy Plus Mini Kit (Qiagen, Germany). Total RNA (1 μg) was reverse transcribed to cDNA using the Quantitect Reverse Transcription Kit (Qiagen). Quantitative real-time PCR was performed using the Top real qPCR 2xPre MIX (SYBR Green with low ROX) with primers that specifically amplify genes for eosinophil cationic protein (ECP), IL-4, IL-13, IFN-γ, IL-17A, CXCL1, CXCL2, CCL11, CCL24, T-box transcription factor (T-bet), GATA3, and RAR-related orphan receptor γ (ROR-γ). Expression of glyceraldehyde-3-phosphate dehydrogenase (*Gapdh*) was used as an internal control for normalization (Table E1). Cycling conditions were 95°C for 10 minutes, followed by 40 cycles at 95°C for 10 seconds, 50°C for 1 minute, and 72°C for 1 minute. Relative gene expression was calculated by using the comparative 2-ΔΔCq method. The PCR efficiency in all runs was close to 100%, and all samples were tested in duplicate.

Quantitative determination of OVA-specific and total IgE in serum and nasal lavage

Blood was collected via cardiac puncture 24 hours after the last intranasal administration, centrifuged, serum removed, and stored at −80°C. Quantitative assessments of OVA-specific IgE and

total IgE in serum and nasal lavage were performed using an enzyme-linked immunosorbent assay (ELISA) kit purchased from BioLegend (San Diego, CA). The sensitivity of OVA-specific IgE and total IgE was 20.7 pg/mL and 0.1 ng/mL, respectively. All procedures were performed according to manufacturer instruction.

References

E1. Kim, D. W. *et al.* Staphylococcus aureus enterotoxin B contributes to induction of nasal polypoid lesions in an allergic rhinosinusitis murine model. *Am J Rhinol Allergy* **25**, e255-261 (2011).

E2. Jacob, A. & Chole, R. A. Survey anatomy of the paranasal sinuses in the normal mouse. *Laryngoscope* **116**, 558-563 (2006).

Online Repository Figure Legends

Fig E1. Representative western blots of cleaved caspase-3 and PARP in EoL-1, THP-1, and RPMI 2650 cells after YC-1 and chaetocin treatment. **A.-C**. Representative western blots of cleaved caspase-3 and PARP in EoL-1, THP-1, and RPMI 2650 cells treated with YC-1 and chaetocin incubated under normoxic and hypoxic conditions for 24 hours (h).

Fig E2. Representative western blots of cleaved caspase-3 in EoL-1, THP-1, RPMI 2650, and THP-1 cells after wogonin treatment. **A.-D**. Representative western blots of cleaved caspase-3 in EoL-1, THP-1, RPMI 2650, and HMC-1 cells treated with wogonin and incubated under normoxic and hypoxic conditions for 16 and 24 hours.

Fig E3. Immunohistochemical observations of survivin expression. **A**. Immunochemical staining of survivin was performed in nasal mucosal tissue from control subjects and patients with CRSsNP, a patient with CRSwNP, and an NP sample. A high number of survivin-positive immune cells was detected in NP tissue (original magnification ×1000). **B**. The percentage of survivin-positive immune cells was significantly greater in the NP tissues. The black box indicates the magnified area. Data are expressed as the means ± SD. *$P < 0.05$, **$P < 0.01$. Mann-Whitney *U* test. Scale bar = 50 μm.

Fig E4. Photographs of representative epithelial disruptions and neutrophil elastase-positive cells in mice. **A**., **B**. The numbers of epithelial disruptions were reduced in Groups C and D compared with that in Group B (Hematoxylin and eosin; original magnification ×400). **C**. Representative photographs and (**D**) quantitative analysis of neutrophil elastase-positive cells (IHC; original magnification ×1000). Data are expressed as the means ± SD. *$P < 0.05$, **$P < 0.01$. Mann-Whitney *U* test. Scale bar = 20 μm.

Fig E5. Cytologic assessment of the nasal lavage and total and OVA-specific IgE production. **A**. Microscopic examination of nasal lavage stained with the standard Diff-Quik protocol. Nasal cytology

shows a predominance of eosinophilic infiltration in Groups B, C, and D. Black arrows point to an eosinophil with apoptotic morphology (original magnification, ×1000, bright field) in Group C. **B., C**. Percentage of apoptotic cells and total inflammatory cells count in each group. **D**.-**G**. The level of total IgE and OVA-specific IgE from serum and nasal lavage measured by enzyme-linked immunosorbent assay (ELISA). Data are expressed as the means ± SD. *$P < 0.05$, **$P < 0.01$. Mann-Whitney *U* test. Scale bar = 20 μm.

Fig E6. TUNEL and survivin-positive cells in a murine nasal polyp model. **A**. Representative photographs of TUNEL and survivin-stained sections and (**B**., **C**) quantitative analysis of TUNEL and survivin-positive cells (original magnification, ×1000, bright field). Arrows denote TUNEL-positive apoptotic cells in the wogonin-treated group. Data are expressed as the means ± SD. *$P < 0.05$, **$P < 0.01$. Mann-Whitney *U* test. Scale bar = 20 μm.

Fig E7. Histopathological evaluation of nasal mucosal tissue. **A**. Representative photographs of Sirius red, Giemsa, periodic acid-Schiff (PAS), Masson' trichrome, and collagen type I (original magnification, ×1000)-stained nasal tissue sections. **B**., **C**. The presence of eosinophils and mast cells were detected in Groups B, C, and D. Group B showed a markedly denser distribution of eosinophils than groups C and D. **E**.-**G**. Comparison of epithelial, subepithelial thicknesses, and collagen type I among the groups. Groups C and D showed less collagen deposition that Group B in sinonasal tissue sections. Data are expressed as the means ± SD. *$P < 0.05$, **$P < 0.01$. Mann-Whitney *U* test. Scale bar = 20 μm.

Fig E8-12. Uncropped Images of Immunoblots

Fig E13. Schematic illustration of the study.

Table E1. qPCR primers used in this study.

**ECP :**	**F: 5’-TCGGAGTAGATTCCGGGTG-3’**<br>**R: 5’-GAACCACAGGATACCGTGGAG-3’**
**IL-4:**	**F: 5’-AGATGGATGTGCCAAACGTCCTCA-3’**<br>**R: 5’-AATATGCGAAGCACCTTGGAAGCC-3’**
**IL-13:**	**F: 5’-TGAGGAGCTGAGCAACATCACACA-3’**<br>**R: 5’-TGCGGTTACAGAGGCCATGCAATA-3’**
**IFN-g:**	**F: 5'-TCCACATCTATGCCACTTGAG-3'**<br>**R: 5'-CTGAGACAATGAACGCTACACA-3’**
**CXCL1:**	**F: 5'-GTGCCATCAGAGCAGTCT-3'**<br>**R: 5'-CCAAACCGAAGTCATAGCCA-3’**
**CXCL2:**	**F: 5'-CTTTCCAGGTCAGTTAGCCTT-3'**<br>**R: 5'-CAGAAGTCATAGCCACTCTCAAG-3’**
**CCL11:**	**F: 5’-TGTAGCTCTTCAGTAGTGTGTTG-3’**<br>**R: 5’-CTTCTATTCCTGCTGCTCACG-3’**
**CCL24:**	**F: 5’-GTACAGATCTTATGGCCCTTCT-3’**<br>**R: 5’-CTGCACGTCCTTTATTTCCAAG-3’**
**IL-17A:**	**F: 5'-CTCCAG AAGGCCCTCAGACTA-3'**<br>**R: R: 5'-AGCTTTCCCTCCGCATTGACA-3'**
**T-bet:**	**F: 5'-CAAGACCACATCCACAAACATC-3'**<br>**R: 5'-TTCAACCAGCACCAGACAG-3'**
**GATA3:**	**F: 5'-GAAGGCATC CAGACCCGAAAC-3'**<br>**R: 5'-ACCCATGGCGGTGACCATGC-3'**
**ROR-g:**	**F: 5'-ACCTCTTTTCACGGGAGGA-3'**<br>**R: 5'-TCCCACATCTCCCACATTG-3'**
**GAPDH:**	**F: 5’-TGCACCACCAACTGCTTAG-3’**<br>**R: 5’-GGATGCAGGGATGATGTTC-3’**

Figure E1

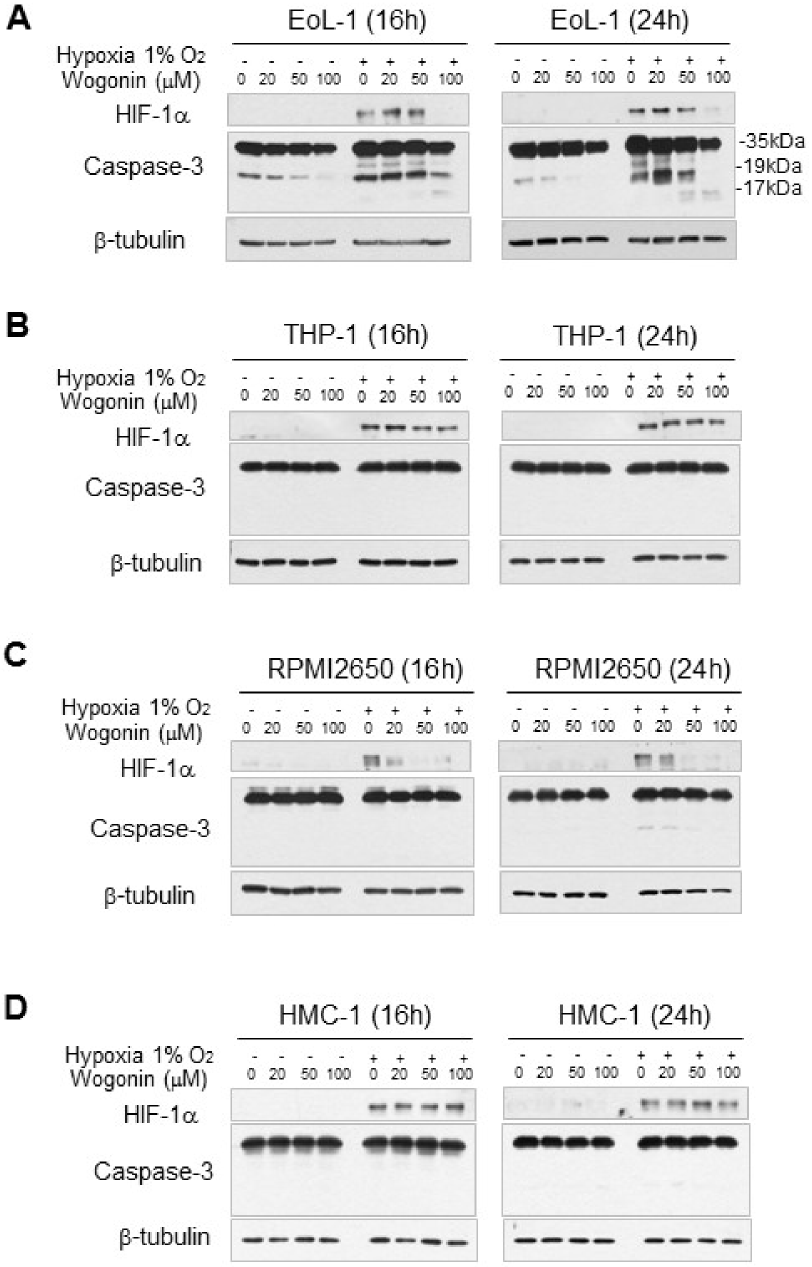
A
EoL-1 (16h)
EoL-1 (24h)
Hypoxia 1% O2
Wogonin (μM)
0 20 50 100 0 20 50 100
HIF-1α
Caspase-3
β-tubulin
-35kDa
-19kDa
-17kDa
B
THP-1 (16h)
THP-1 (24h)
C
RPMI2650 (16h)
RPMI2650 (24h)
D
HMC-1 (16h)
HMC-1 (24h)

Figure E2

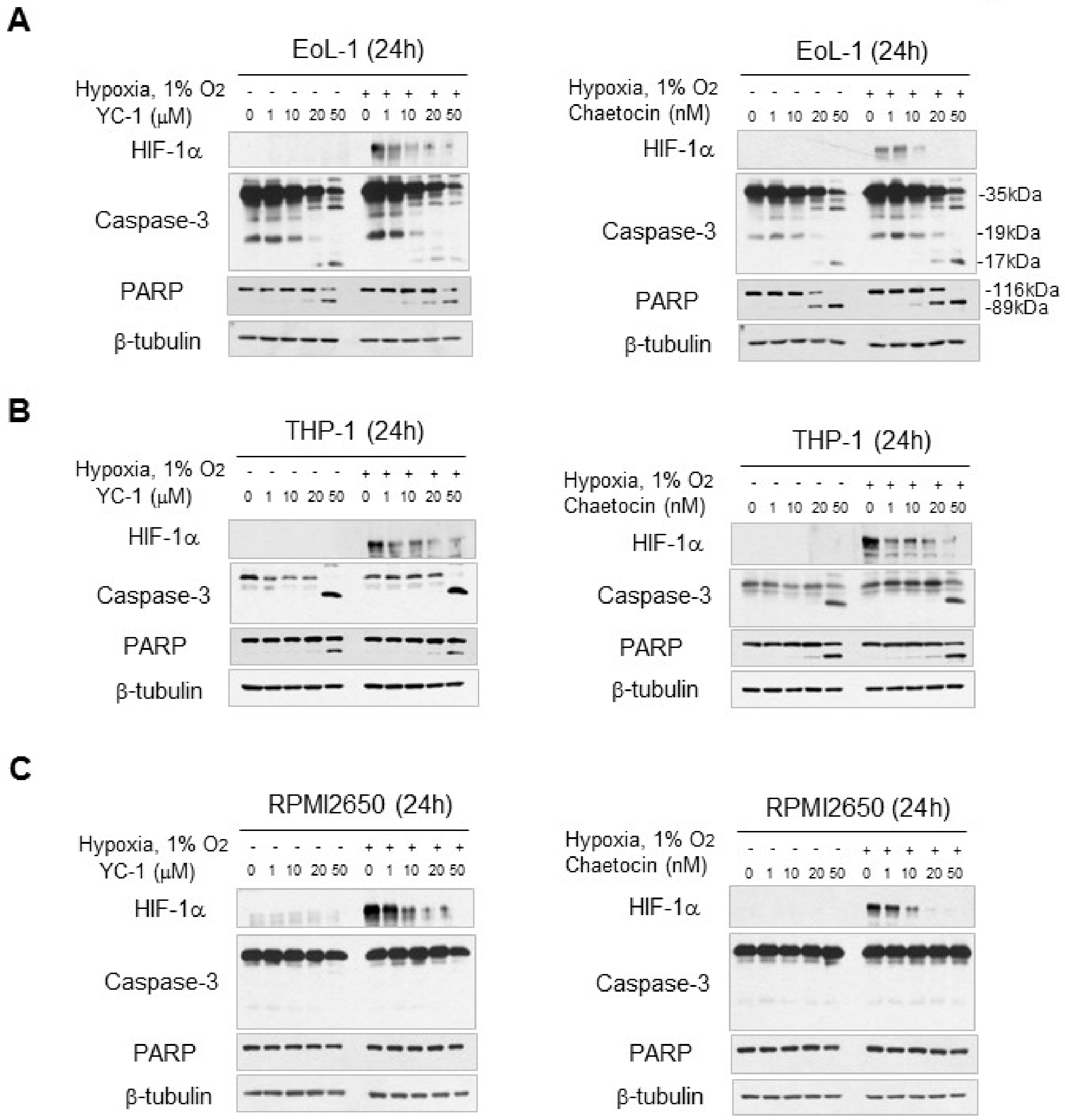

199

200

Figure E3

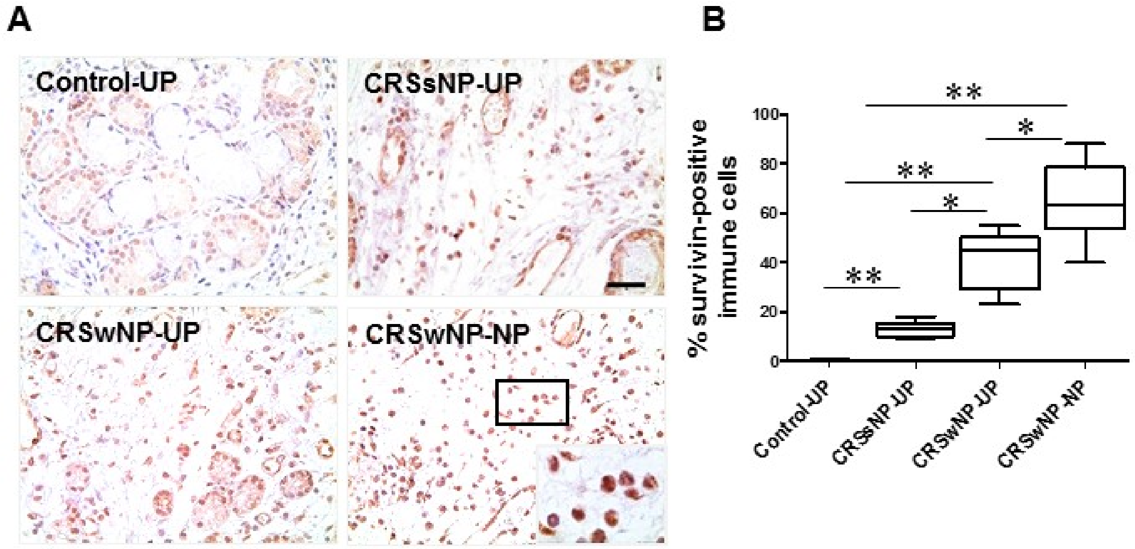

201

202

Figure E4

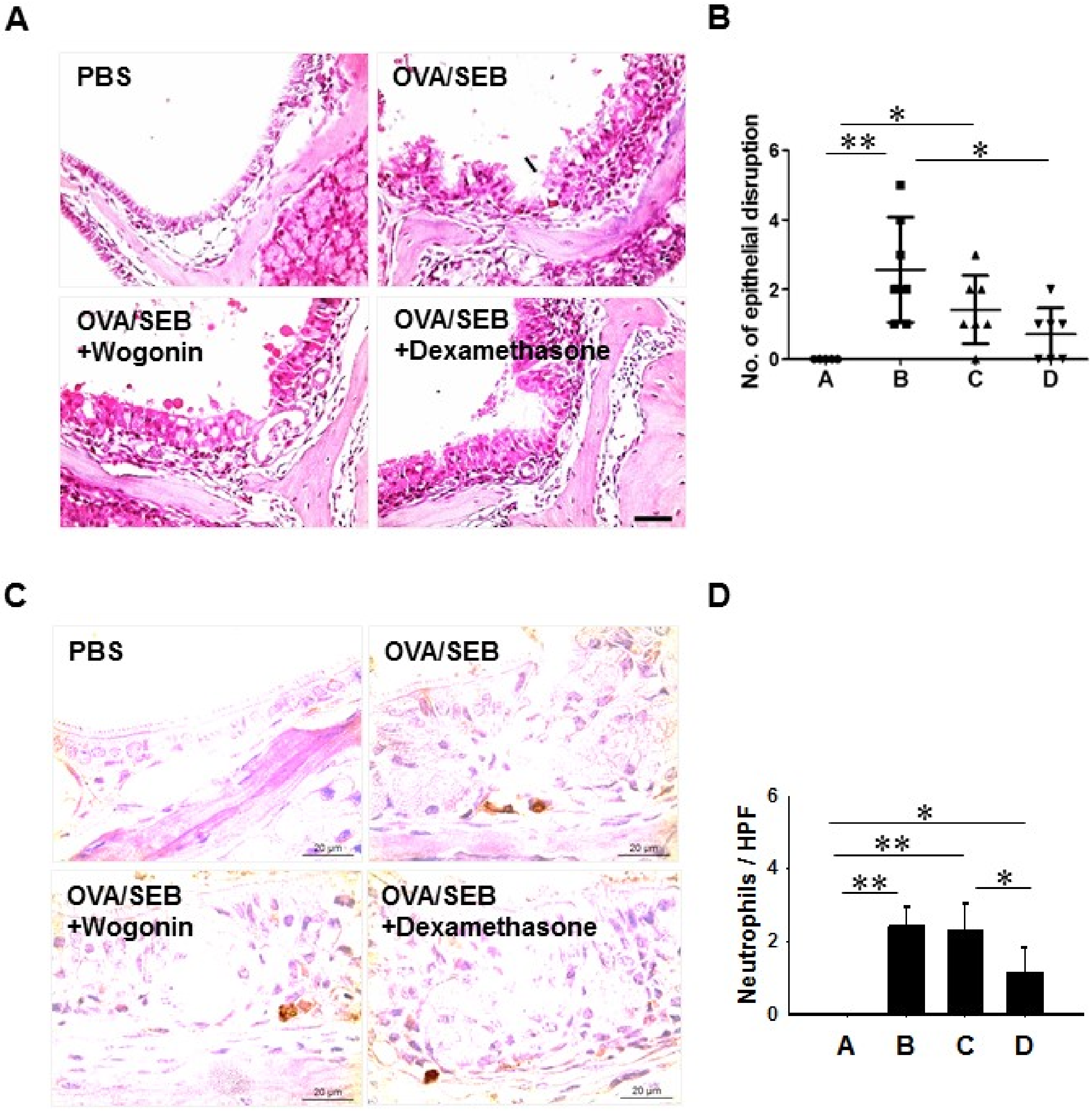
A
PBS
OVA/SEB
OVA/SEB
+Wogonin
OVA/SEB
+Dexamethasone
B
No. of epithelial disruption
0
2
4
6
A
B
C
D
**
*
*
C
PBS
OVA/SEB
OVA/SEB
+Wogonin
OVA/SEB
+Dexamethasone
D
Neutrophils / HPF
0
2
4
6
A
B
C
D
*
**
**
*

Figure E5

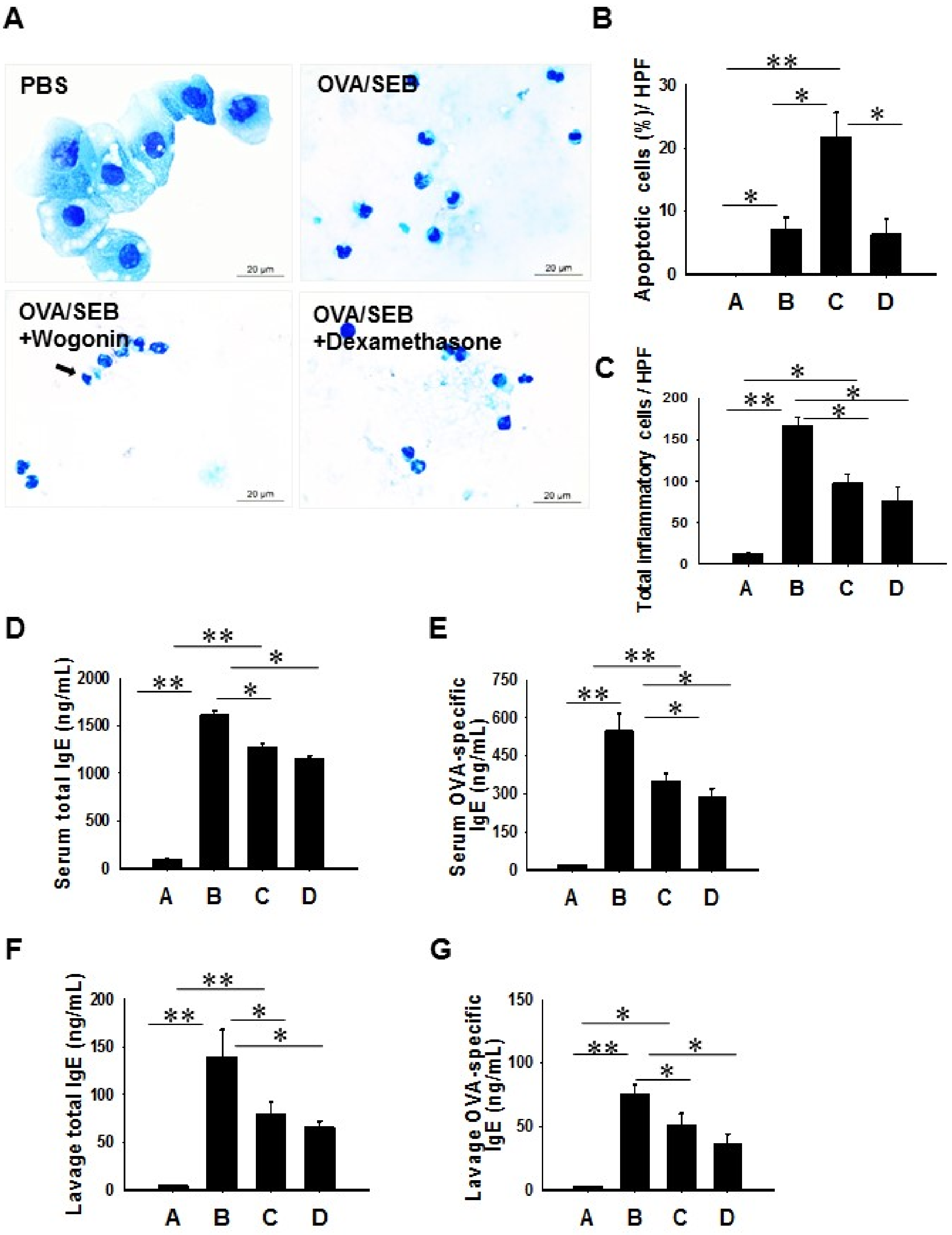

205

206

207

Figure E6

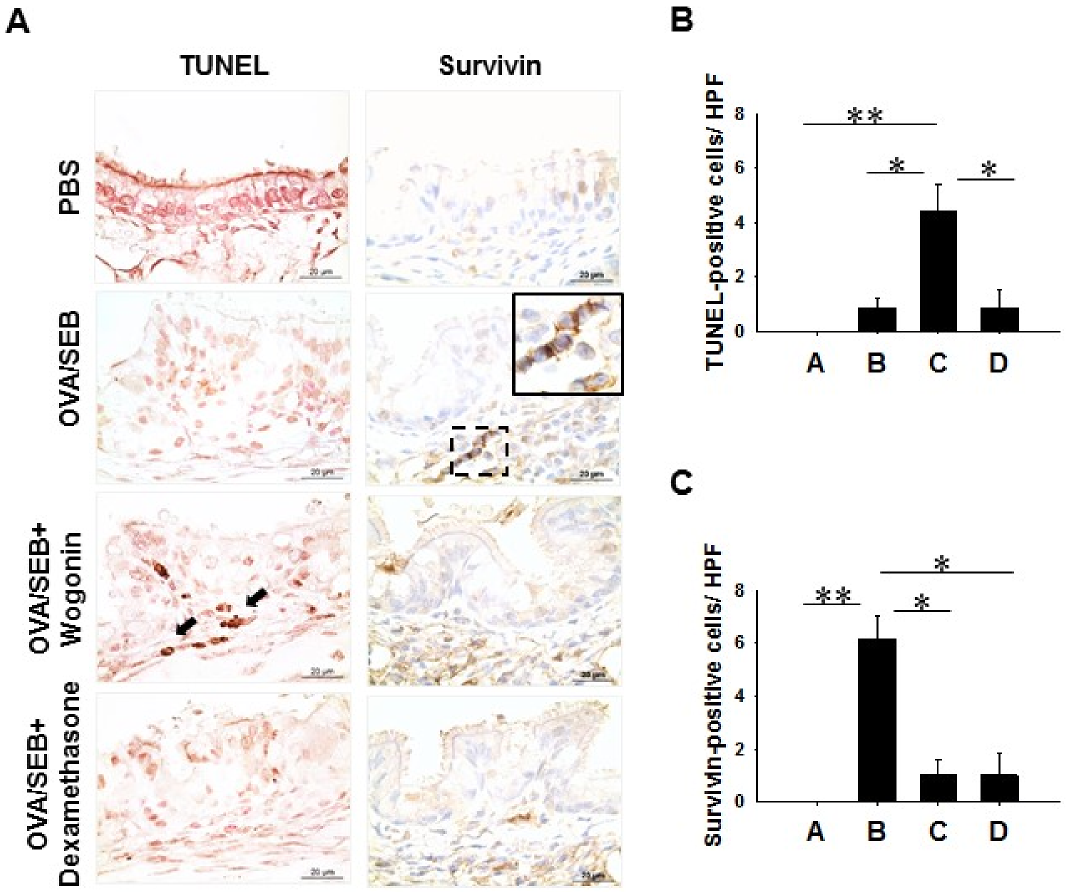

208

209

Figure E7

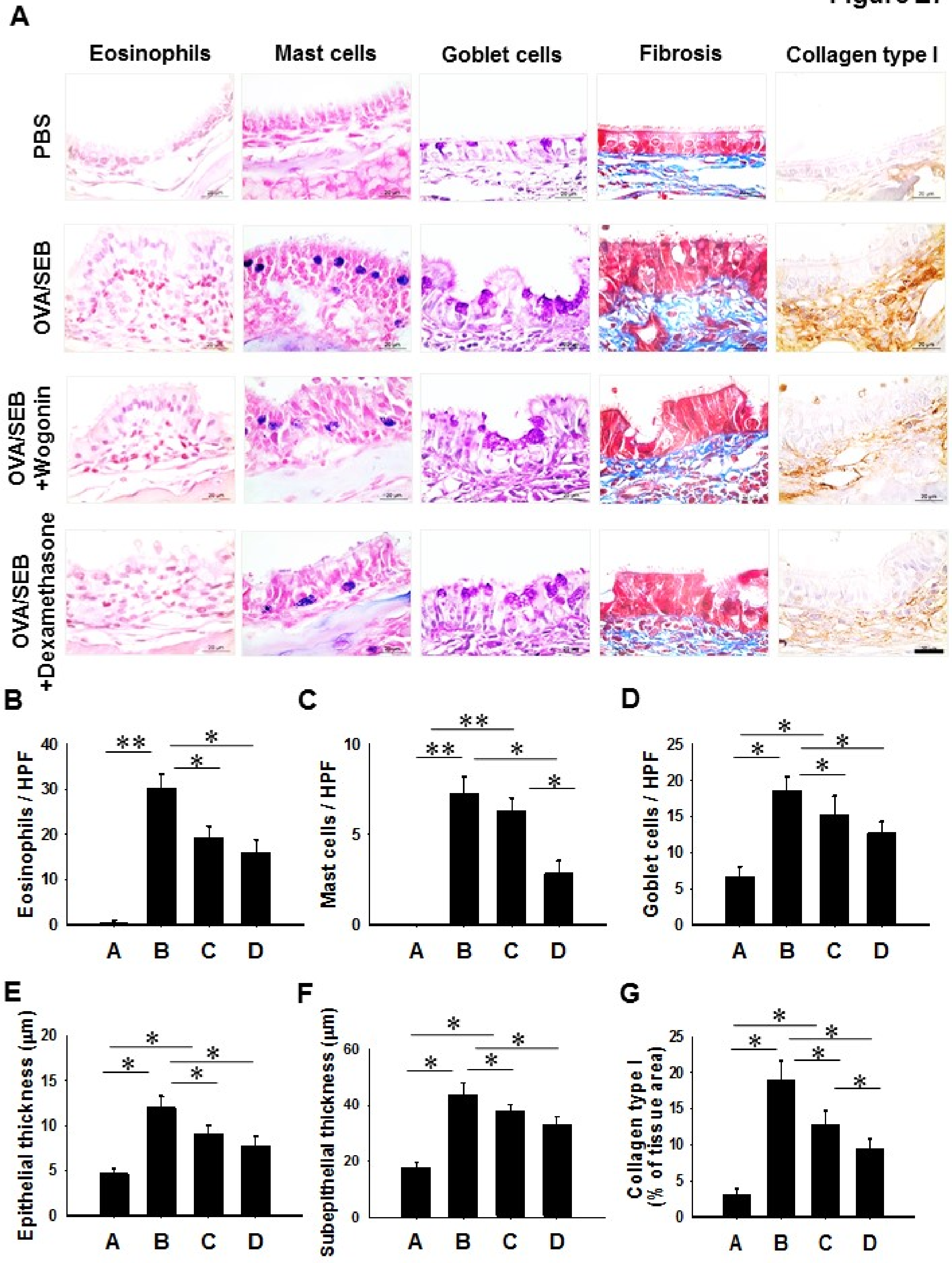

210

211

Figure E8

Fig 2A

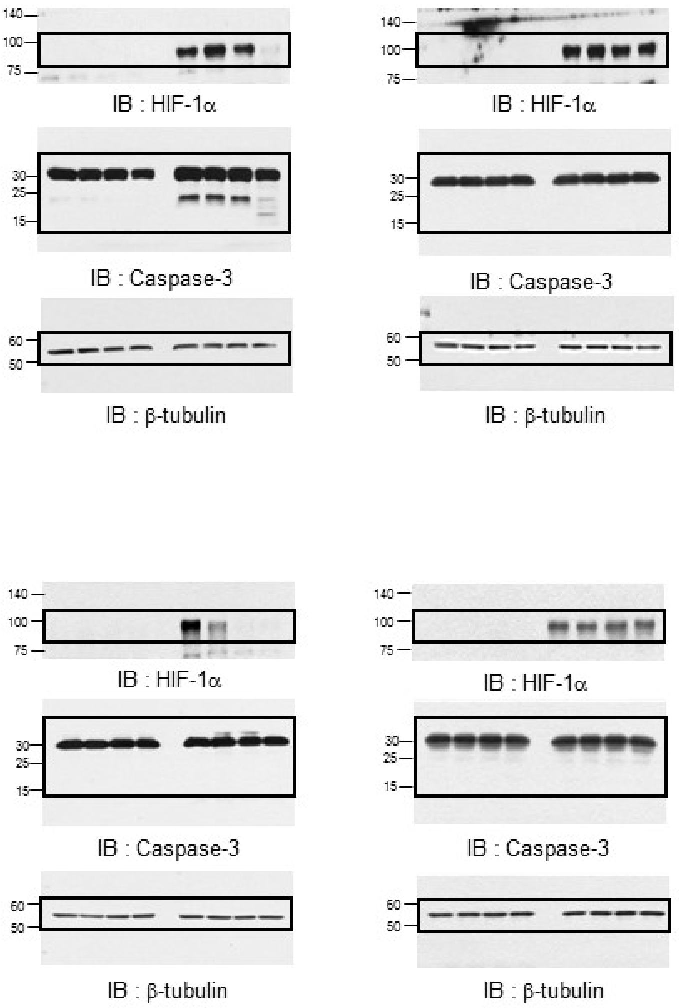

Figure E9

Fig 3A

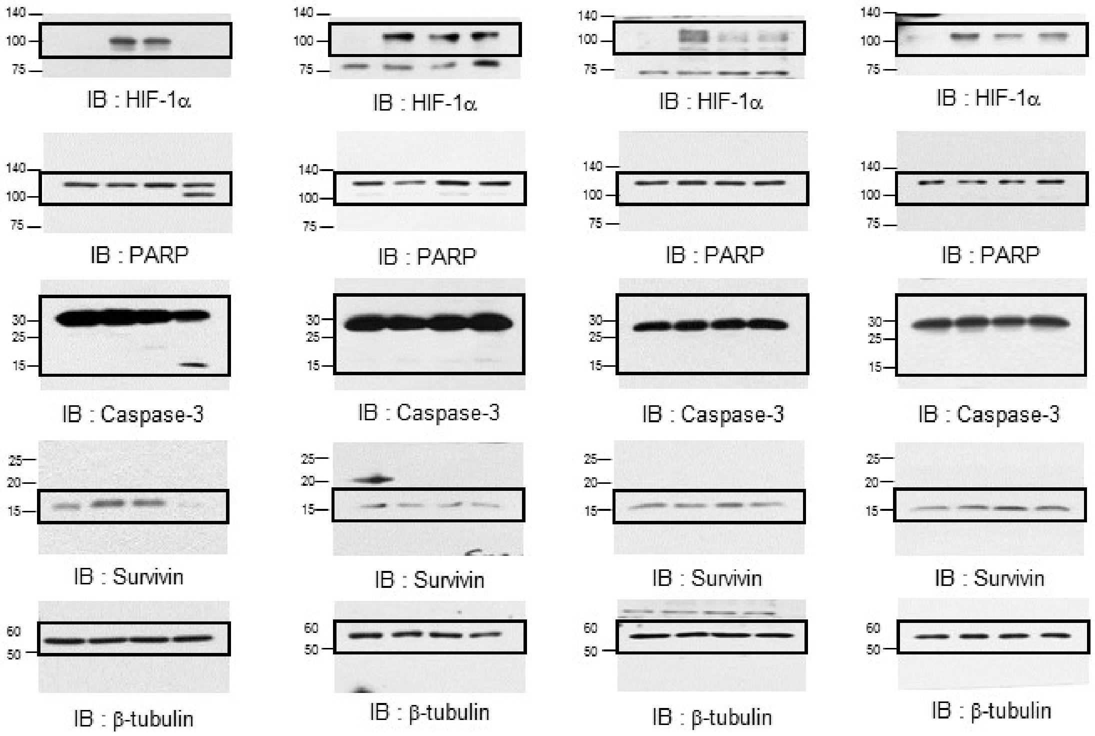

Figure E10

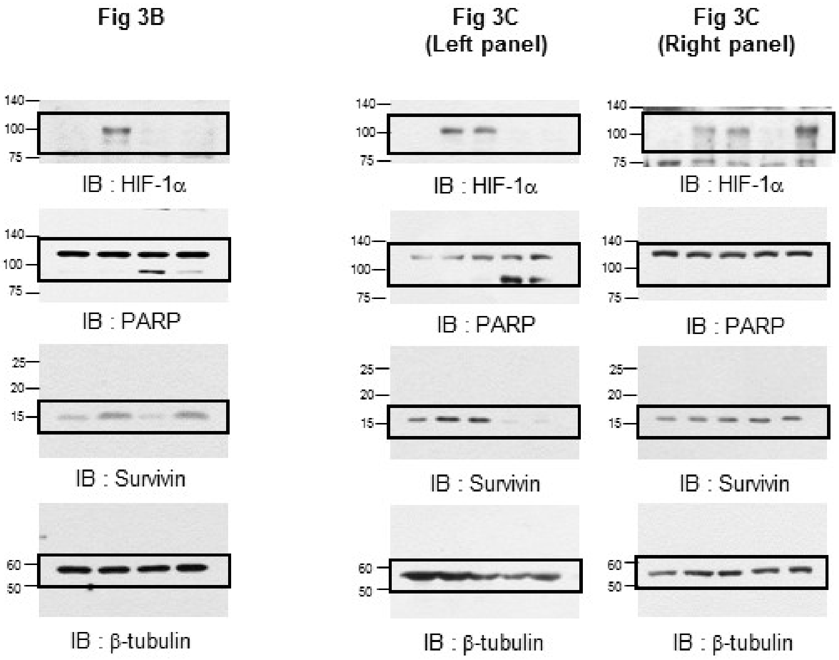
Fig 3B
Fig 3C
(Left panel)
Fig 3C
(Right panel)
140
100
75
IB : HIF-1α
IB : PARP
25
20
15
IB : Survivin
60
50
IB : β-tubulin

Figure E11

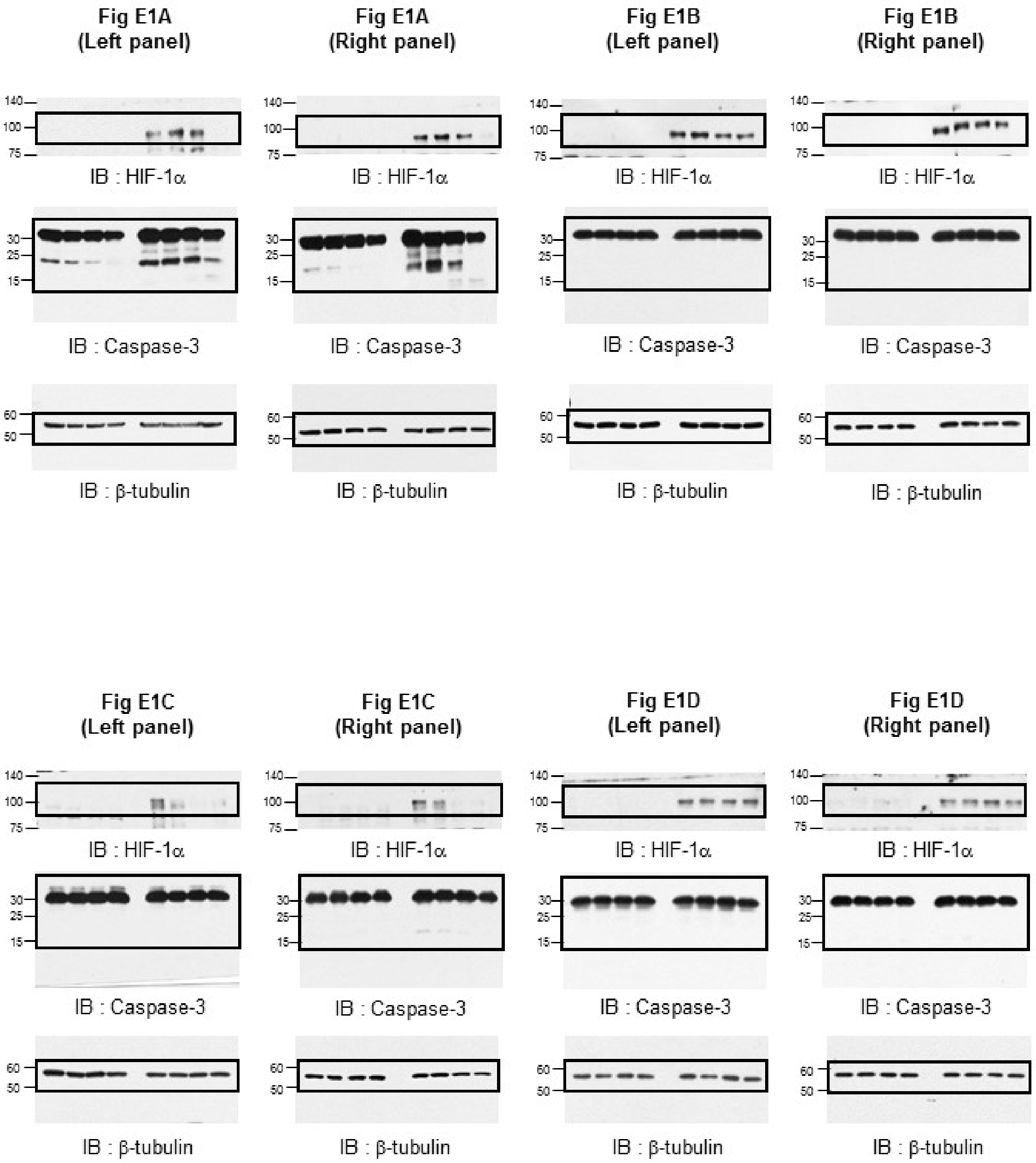
Fig E1A
(Left panel)
Fig E1A
(Right panel)
Fig E1B
(Left panel)
Fig E1B
(Right panel)
IB : HIF-1α
IB : Caspase-3
IB : β-tubulin
Fig E1C
(Left panel)
Fig E1C
(Right panel)
Fig E1D
(Left panel)
Fig E1D
(Right panel)
140
100
75
30
25
15
60
50

Figure E12

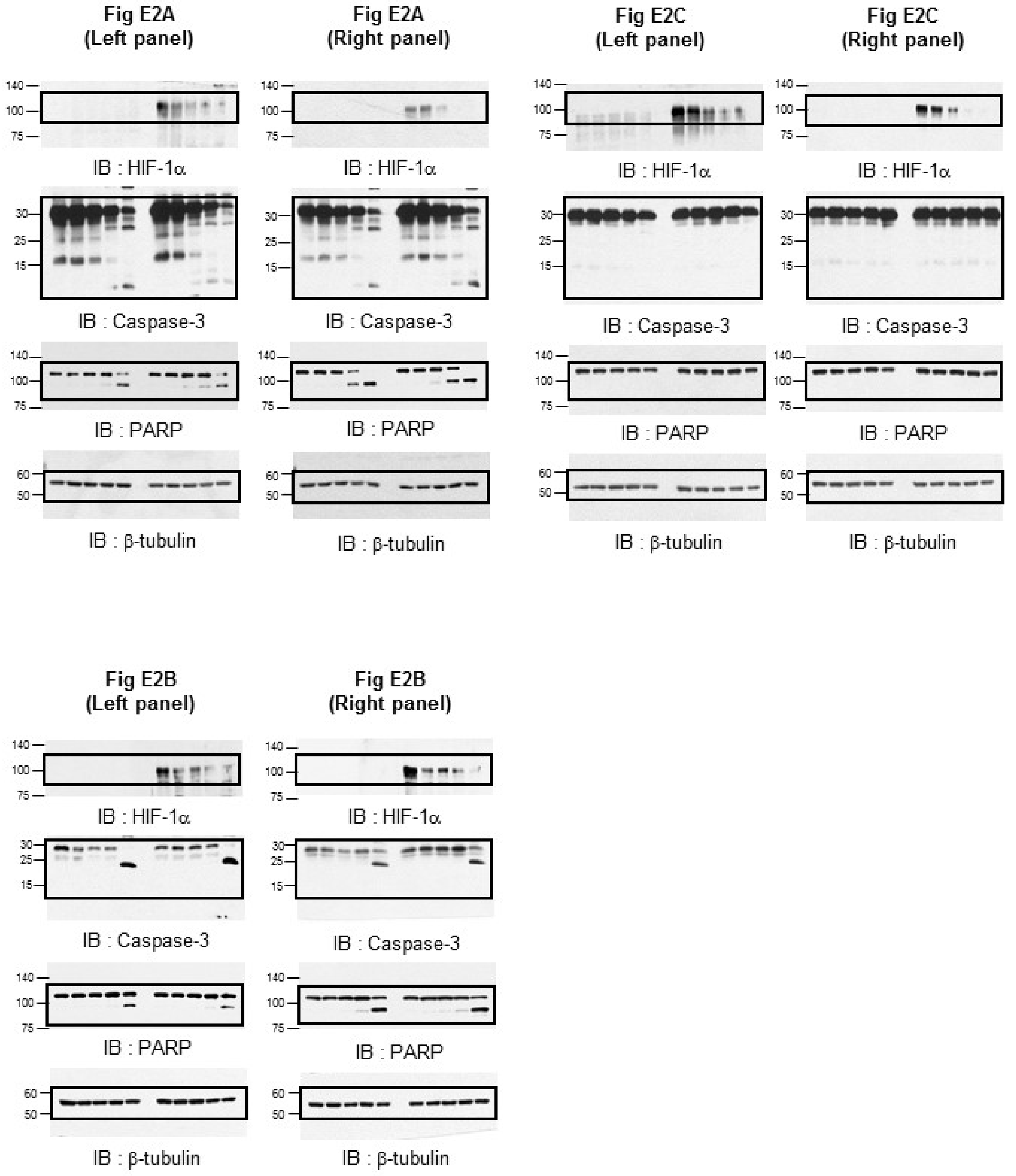

Fig E2A (Left panel)
Fig E2A (Right panel)
Fig E2C (Left panel)
Fig E2C (Right panel)
140
100
75
IB : HIF-1α
30
25
15
IB : Caspase-3
IB : PARP
60
50
IB : β-tubulin
Fig E2B (Left panel)
Fig E2B (Right panel)

Figure E13

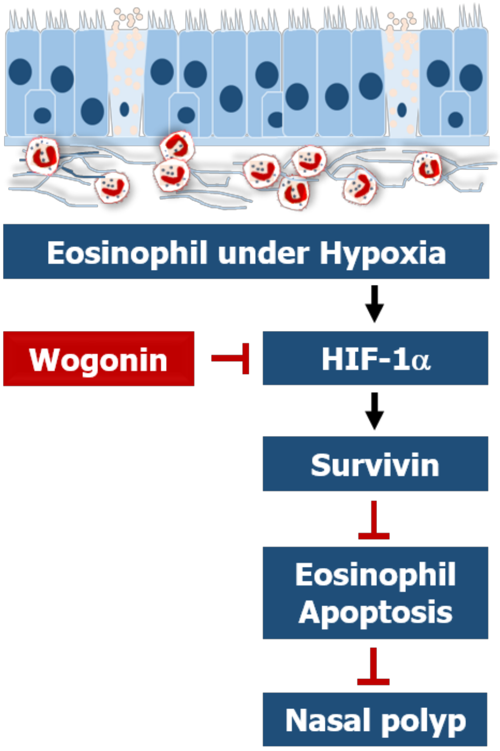
Eosinophil under Hypoxia
Wogonin
HIF-1α
Survivin
Eosinophil
Apoptosis
Nasal polyp
